# Perceptions, perspectives and experiences of adult patients attending nurse-led clinics: a mixed-method systematic review

**DOI:** 10.1186/s12912-026-04740-7

**Published:** 2026-05-20

**Authors:** Dhanya Varghese, Samara J. Geering, Souti Khalil, Yashwani S. Lata, Sandra J. Sullivan, Nicole J. Weekes, Sheeja P. Pathrose, Josephine Sf Chow, Marida Ritha, Gladis Kabil

**Affiliations:** 1 Clinical Nurse Consultant – Cardiology, Bankstown Lidcombe Hospital, South Western Sydney Local Health District (SWSLHD), Bankstown, NSW Australia; 2 South Western Sydney Nursing & Midwifery Research Alliance, SWSLHD, Liverpool, NSW Australia; 3 Campbelltown and Camden Hospital, SWSLHD, Campbelltown, NSW Australia; 4 Liverpool Hospital, Liverpool, NSW Australia; 5 Primary and Community Health, SWSLHD, Liverpool, NSW Australia; 6 Lymphoma Australia, Brisbane, Queensland Australia; 7 School of Nursing and Midwifery, Western Sydney University, Locked Bag 1797, Penrith, NSW 2751 Australia; 8 JBI Western Sydney Centre, University of Adelaide, Penrith, NSW Australia; 9 Ingham Institute for Applied Medical Research, Liverpool, NSW Australia; 10 Faculty of Medicine, University of New South Wales, Camperdown, NSW Australia; 11 School of Nursing and Midwifery, University of Tasmania, Lilyfield, NSW Australia; 12 Westmead Hospital, Western Sydney Local Health District, Westmead, NSW Australia

**Keywords:** Nurse-led clinics, Patient perception, Patient experiences, Patient satisfaction, Advanced practice nurses, Acceptability of the care, Care and support, Accessibility

## Abstract

**Background:**

Nurse-led clinics (NLCs) are structured models of care in which nurses assume primary responsibility for patient care, often practising autonomously within multidisciplinary teams across all healthcare settings. Developed to address rising clinical demands, workforce shortages, and the growing chronic disease burden, NLCs play a key role in delivering accessible, holistic, patient-centred care led by advanced practice nurses. Although a qualitative systematic review of patient experiences with NLCs was conducted in 2012, evolving models of care and research designs highlight the need for updated evidence. Accordingly, this mixed-methods systematic review employs a narrative synthesis approach to examine and synthesise the perceptions, perspectives, and experiences of adult patients attending NLCs, including factors that influence these perceptions.

**Methods:**

Published research from 2012 to April 2025 was retrieved using the electronic databases MEDLINE, CINAHL, Embase, Scopus, and PsycINFO using a comprehensive search strategy. All articles were collated in EndNote and screened via COVIDENCE by two independent reviewers. Data were extracted from the articles that met the inclusion criteria using a standardised form. Quality appraisal was undertaken using JBI critical appraisal tools.

**Results:**

A total of 3185 articles were identified of which 23 studies met the inclusion criteria were included in the final synthesis. A mix of quantitative and qualitative methodologies were applied in these articles, with 12 using a cross-sectional design with a structured patient satisfaction survey, seven of these studies employed a qualitative approach while four studies used a mixed method approach. Key findings identified include patient satisfaction with the length of consultation time, the confidence in the care provided by the APN, decreased waiting times, improved confidence in managing disease, continuity of care, person centred approach and the feeling of being involved in the decision making. NLCs generally met patient expectations, with patients reporting satisfaction, accessibility, increased knowledge, timely access, service acceptability, and holistic care.

**Conclusion:**

This review demonstrated that patients highly value nurse-led clinics across diverse healthcare settings for their accessibility, continuity, timeliness, and the holistic care provided by APNs. These insights can guide future service improvements and enhance patient experiences.

**Clinical trial number:**

Not applicable.

**Supplementary Information:**

The online version contains supplementary material available at 10.1186/s12912-026-04740-7.

## Background

An ageing population has resulted in people living longer, with more complex and chronic health conditions [1]. The demand for clinical services continues to grow exponentially, increasing workload and strain on medical practitioners and challenging the health care system [2]. A solution to these increased service demands since the 1980s has been the development of NLCs. They are an alternative to Medical Practitioner-led clinics and were initially developed to bridge gaps, expand healthcare coverage, and, in response to growing clinical demand and healthcare workforce scarcity, promote health equity [3].

Nurse-led clinics (NLCs) are an alternative model of health care delivery in which nurses are the primary providers of care [4]. While there is no uniform definition across the literature, Wong and Chung [5] define NLCs as a formalised, structured mode of healthcare delivery involving a nurse and a client. In this model, nurses may work independently or collaboratively with other members of the multidisciplinary healthcare team. Today, NLCs are operational across primary, secondary, and tertiary healthcare settings worldwide.

The general consensus is that nurses have their own caseload in NLCs, increased autonomy and greater responsibility for patient care and management [6, 7]. Nurses who practise within NLCs generally perform health care actions that others may be uncomfortable with or unable to perform. In addition, nurses may adopt key elements of other health professionals’ work [8]. NLCs are commonly performed with the assistance of a multidisciplinary team, in which nurses conduct a variety of assessments and coordinate patient care, such as medication administration, patient education, and referral to allied health or medical practitioners. [1]

Nurse-led clinics are commonly structured around established health-system frameworks, such as the WHO Integrated People-centred Health Services Framework [9], McCormack & McCance, Person Centred Nursing Framework [10], which emphasises coordinated, person-centred, team-based approaches to delivering high-quality care, and the interrelationship between practitioners’ attributes, care environments, and outcomes. As the nursing profession has evolved and responded to increased service demands, many nurses have transitioned to advanced practice nursing roles, functioning autonomously, and employing advanced health assessment and intervention skills [5]. NLCs are generally managed by Advanced Practice Nurses (APNs) [7]. The Nursing and Midwifery Board of Australia [11] defines APNs as nurses who incorporate professional leadership, education, research, and support of systems into their practice. Their practice includes relevant expertise, critical thinking, complex decision-making, and effective, safe autonomous practice. APNs offer a holistic and patient-centred approach to care, enhancing patient engagement and satisfaction [7].

While the literature extensively documents the outcomes of NLCs from clinical and health service perspectives, there is comparatively limited exploration of the patient experience, particularly from a qualitative perspective. Nurse-led models of care have been shown to be beneficial, safe, and cost-effective approaches to healthcare. In turn, we continue to see the implementation of NLC models worldwide, along with growing recognition of the benefits these models provide [2, 12]. Given the person-centred nature of nursing, it is important to understand how this approach influences patients’ perceptions of care within NLCs [13].

In 2012, a qualitative systematic review by Jakimowicz [14] examined studies published between 1990 and 2012 on factors that impact patients’ subjective experiences and perceptions of NLCs. This review highlighted the strong alignment between NLCs and person‑centred care, identifying key elements such as respect for individual patient needs, including beliefs, cultural background, and personal values. The findings also highlighted the importance of mutual trust and respect, attention to psychosocial factors, and acknowledgement of patients’ choices and preferences, while fostering autonomy, empowerment, and holistic care within a guided and supportive model. However, the review only included qualitative studies. Jakimowicz et al. [13], focus on the person-centred nature of nurse-led clinics and the factors that impact patients’ subjective experiences [15]. The demand for healthcare workers has grown due to an aging population, high rates of chronic disease, increasing consumer expectations, and a reliance on short-term medical interventions. As a result, demand for healthcare workers, such as nurses, is expected to continue rising in order to meet increasing care needs [16]. Consequently, as the nursing workforce and nurse-led clinics have evolved, several studies have used both qualitative and quantitative approaches to evaluate NLCs; therefore, using a mixed-methods approach to synthesise the evidence will provide high-quality insights into patient experiences, satisfaction, and confidence in NLCs. This review was not conducted as an update of the previous systematic review done in 2012, as the aim of this review was to capture broader evidence without excluding studies based on their design. The review was undertaken as a mixed-methods synthesis integrating quantitative and qualitative evidence to capture contemporary developments in nurse-led clinic models. This will help guide service improvements and support nurse-led models of patient-centred healthcare delivery. This study prioritises relevance to current health care practice and service delivery.

The purpose of this mixed-method systematic review is to examine the perspectives, perceptions, and experiences of adult patients attending nurse-led clinics.

## The review

### Definitions

For the purposes of this study, the term *nurse-led clinic (NLC)* is defined in accordance with Terry et al. [8] as a structured model of healthcare delivery involving direct interaction between a nurse and a client. The “nurse-led” term illustrates the broader scope of practice, in which they perform healthcare tasks that may exceed the standard scope of general nursing practice. These nurses often possess advanced competencies in specialised areas, enabling them to provide expert care within a clearly specified clinical framework [7]. APNs operating in these clinics exercise an elevated level of clinical autonomy and independent decision-making to address complex patient needs [7].

NLCs adopt a holistic and person-centred approach, aiming to enhance patient experiences and clinical outcomes [8]. This approach is associated with increased patient satisfaction and engagement, as APNs deliver care that is both clinically effective and tailored to individual needs [7]. In this study, patient satisfaction is defined as the subjective effectiveness of care received, considering factors other than clinical outcomes. This includes elements such as the professionalism of the staff, timeliness of the services, acceptability, and accessibility of the clinic [17, 18].

### Aim

To examine and understand the perceptions, perspectives and experiences of adult patients attending NLCs, including factors that may impact on patient perception.

### Design

A systematic review was conducted based on a priori protocol that was registered in Prospero Review Registration: CRD420250653831.The review is reported using the Preferred Reporting Items for Systematic reviews and Meta-Analyses (PRISMA) 2020 checklist [19]. Our systematic review utilised a mixed methods approach (quantitative and qualitative research). We have utilised JBI methodological guidance for conducting this mixed-method Systematic Review. A systematic review provides a comprehensive understanding of the current research landscape and available evidence [20, 21]. A mixed-method systematic review offers broader insights into the phenomenon under investigation, including patient perceptions and experiences, by incorporating data from qualitative, quantitative, and mixed-method studies [22, 23]. In this review, we adopted a narrative synthesis approach described by Popay et al. [21] for the synthesis of findings from both the quantitative and qualitative studies.

### Eligibility criteria

**Inclusion Criteria**: Qualitative, quantitative, and mixed-method studies that met the predetermined eligibility criteria were included in this review. Primary research focusing on nurse-led health care clinics, ranging inpatient, outpatient, and community-based settings, was considered. Studies included adult patients aged 16 years and older who presented to nurse-led health care clinics. Only articles published in English between 2012 and 2025 were reviewed to include all articles published since the last systematic review conducted in 2012. Eligible studies clearly identified patient perception and experiences as their primary phenomenon of interest. Where studies explored both patient and staff experiences were explored, only data pertaining specifically to patient experiences were extracted.

**Exclusion Criteria:** Research focused on drug health, mental health, and sexual health clinics was not considered due to the significant difference in the model of care used in these settings and the variations in the scope of practice of mental health nurses. Studies investigating clinics operated by midwives or medical consultants, as well as those examining telehealth services, telephone follow-ups, virtual clinics, or nurse-led initiatives outside a clinic setting, were also excluded. As the aim of this study is to explore patient perceptions and experiences of NLCs, where building rapport and trust is a vital component of care, studies exploring telehealth services, telephone follow-ups, and virtual clinics were excluded. Evidence suggests that establishing these interpersonal relationships is challenging in telehealth settings [24]. Patients have reported concerns about difficulty forming provider-patient relationships, feeling less involved during visits, and having fewer opportunities to speak compared to in-person consultations [25]. Additionally, telehealth may exacerbate inequities and pose barriers for individuals living with disabilities or from culturally and linguistically diverse backgrounds [26]. Since this review focuses entirely on patient perceptions and satisfaction with the traditional nurse-led clinic model, including telehealth would introduce a fundamentally different care modality and compromise the scope of the study.

Articles that included staff perceptions, regardless of whether the staff members were medical or nursing staff, were excluded. Additionally, systematic reviews, literature reviews, pilot studies, feasibility studies, and inappropriate interventions were also excluded from the final selection.

The articles selected were based on the PICO/PICO framework (Table 1) in accordance with the JBI mixed-methods methodology [27].

**Table 1:** PICO/PICo framework for article selection

Participants (P)	Patients over the age of 16 years attending a nurse-led clinic.
Phenomena of Interest (I)	Perspectives, perceptions and experiences of adult patients attending a nurse-led clinic. Factors that impact on patient’s subjective experiences.
Intervention (I)	Adults who have received care in NLCs.
Comparator (C)	N/A
Outcome (O)	Perspectives, perceptions and experiences of adult patients attending a nurse-led clinic. Factors that impact on patient’s subjective experiences.
Context (Co)	Nurse-led clinic. We did not place any geographical restrictions.

### Search methods

Studies published between 2012 and 2025 were included. The search for articles began on 4th March 2025 and concluded on 16th April 2025. The electronic databases MEDLINE, CINAHL, Embase, Scopus, and PsycINFO were searched to ensure that literature from both developed and developing nations was adequately captured. In addition, a grey literature search was undertaken using Google Scholar, which identified one relevant thesis, which was included in this review. The final results were then examined for inclusion criteria. Search terms were developed by the team in consultation with an experienced hospital librarian. The search terms used keywords such as “Nurse-led clinics”, “Nurse-led”, “Practice Patterns Nurses”, “Practice Patterns”, “Nurse Managed Centre”, “Nurse Managed Centers”, “Patient experience”, “Patient perception”, “Patient Satisfaction”. The selected search terms were further enhanced through truncation, MeSH headings, and Boolean operators to ensure an optimal and effective search was conducted. The detailed search strategy is included in Appendix 1.

### Data extraction

Articles that met the inclusion criteria were extracted into a pre-developed extraction tool. The extraction tool included the year of publication, country, methodology, participant characteristics, descriptions of NLCs, sample size, and results. Covidence was used to screen and process the articles, and each article was independently reviewed by two reviewers. Conflicts were resolved through an independent third reviewer. Cohen’s K statistic for abstract and title screening showed an inter-rater agreement of 0.732, indicating substantial agreement between reviewers [28].

The data extraction process was guided by a preestablished research question to ensure the consistency and appropriateness of the extracted information [21]. Both qualitative and quantitative methodologies identified in the included studies were considered. A convergent integrated approach, as detailed in JBI evidence synthesis, was utilised for data extraction, as the primary quantitative studies included were cross-sectional surveys [22]. A structured Excel spreadsheet was developed to facilitate systematic and organised data collection across all included studies. Key data items extracted included study characteristics (e.g., citation year, country), methodology (e.g., design, sample size, setting), inclusion and exclusion criteria, primary outcomes, qualitative and statistical analyses, key findings, and conclusions relevant to the research question.

Each researcher independently extracted data from the eligible studies to reduce bias and enhance credibility. Following independent extraction, the team held meetings to compare data and resolve disparities. Disagreements were thoroughly discussed in the consensus meeting and resolved through concerted effort. A third reviewer was consulted to achieve consensus. This stringent approach helped maintain the integrity and explicitness of the data extraction phase.

### Data synthesis

The studies included use a mix of qualitative and quantitative methodologies, which necessitates a narrative synthesis to integrate their findings. This is because the primary quantitative studies consisted of cross-sectional surveys, which do not provide sufficient data to calculate standardised effect sizes [21].

Narrative synthesis is an approach used in systematic reviews to synthesise findings from multiple studies (qualitative, quantitative, and mixed methods), relying on language to summarise and explain the findings. There are four main elements in the narrative synthesis: the role of theory in evidence synthesis, developing a preliminary synthesis, exploring relationships within and between studies, and assessing the robustness of the synthesis [21]. In this study, the elements of our synthesis included, but were not limited to, developing a preliminary synthesis, exploring the relationships in the data, and assessing robustness [21].

**Fig. 1:**
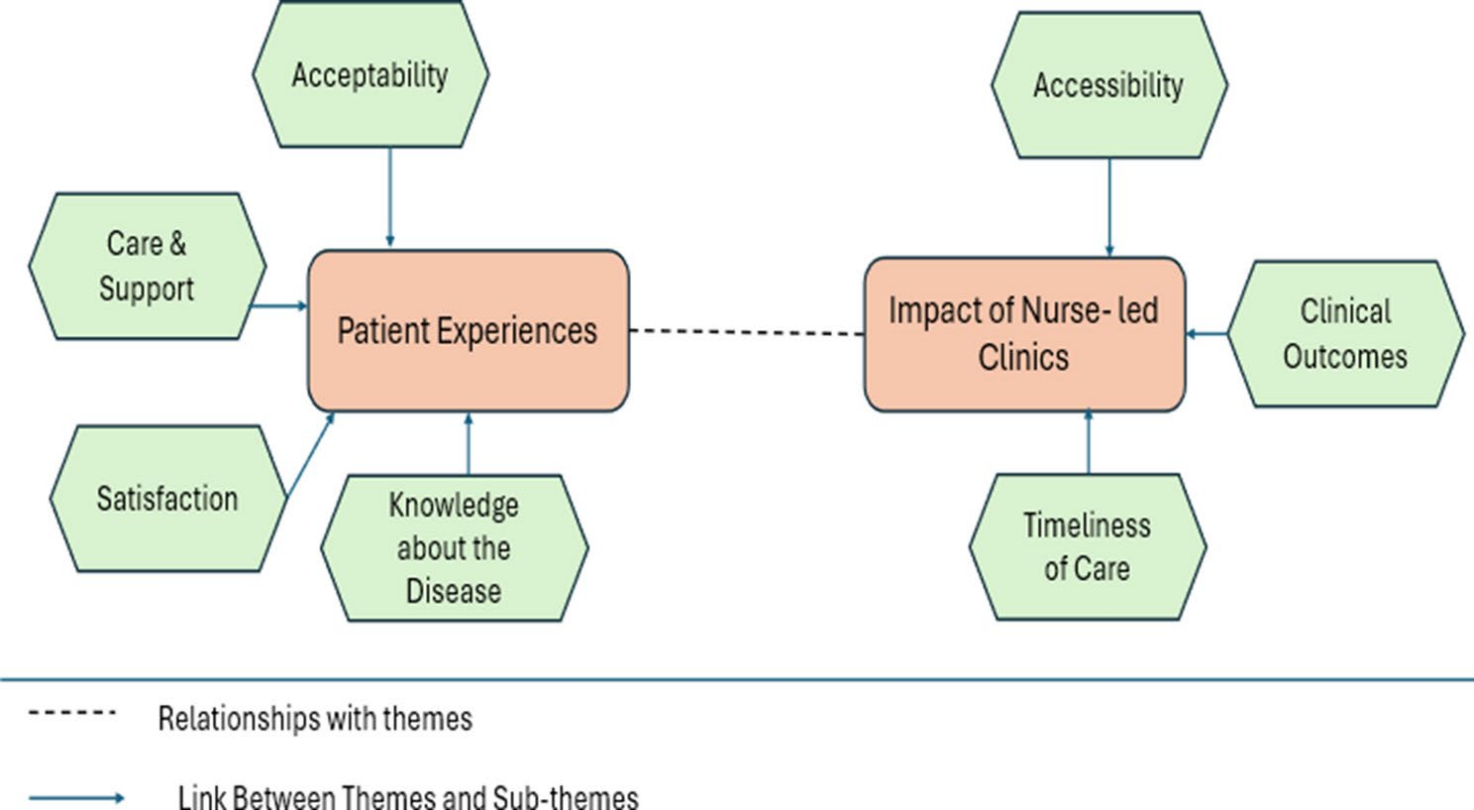
Concept mapping of relationship between themes and subthemes

Narrative synthesis enables the presentation of results clearly and accessibly, particularly when reviewing mixed-methods studies [29]. Two reviewers independently screened and appraised all studies, including mixed-method studies that reported both quantitative and qualitative data. Following the appraisal, the synthesis process was guided by Popay et al.’s [21] framework. Quantitative findings were summarised and interpreted narratively to reflect their relevance to patient experiences. These were then integrated with qualitative findings during thematic analysis to generate overarching themes. Qualitative findings and narrative quantitative findings were analysed together to identify patterns, similarities, and differences across studies [22]. After initial independent coding of themes and subthemes, we developed a preliminary synthesis of the included studies during which the research team met to identify and agree on key themes and subthemes through group consensus. These themes and sub-themes are summarised in Table 2. The interrelationships among the themes and sub-themes are presented in Fig. 1

**Table 2:** Mapped themes & quality of studies

Authors	Satisfaction with the nurse-led clinic	Knowledge about the disease	Care & support	Acceptability	Accessibility	Clinical Outcomes	Timeliness of care	Quality of the studies	
								Cross sectional	Qualitative
Bala et al., 2012			X						80%
Bennett-Daly et al.,2021			X					75%	90%
Berglund et al.,2015			X					100%	
Coleman et al.,2017				X	X			87.5%	
Drewry et al., 2012				X				12.5%	
Fishburn and Fishburn, 2021	X			X			X	25%	
Gyldenvang et al., 2022		X	X	X				75%	90%
Habibi et al.,2023		X		X				75%	80%
Hicks et al.,2012				X		X		37.5%	
Ibrahim et al., 2019	X					X		75%	
Kor et al.,2022	X	X						75%	
Larsson et al.,2012			X	X	X				80%
Momoh et al., 2024	X							87.5%	
Nguyen et al.,2022							X	25%	
Petrushnko et al.,2024			X		X			37.5%	
Pun et al.,2023			X	X	X				80%
Ramachandran et al., 2022		X							80%
Sjö and Bergsten, 2018		X		X					100%
Stirling et al.,2016				X					80%
Taylor et al., 2018			X						80%
Vanalia et al., 2023		X	X	X			X	75%	80%
Williams et al.2012				X				75%	
Winter et al.,2012	X			X				37.5%

## Results

### study characteristics

**Fig. 2:**
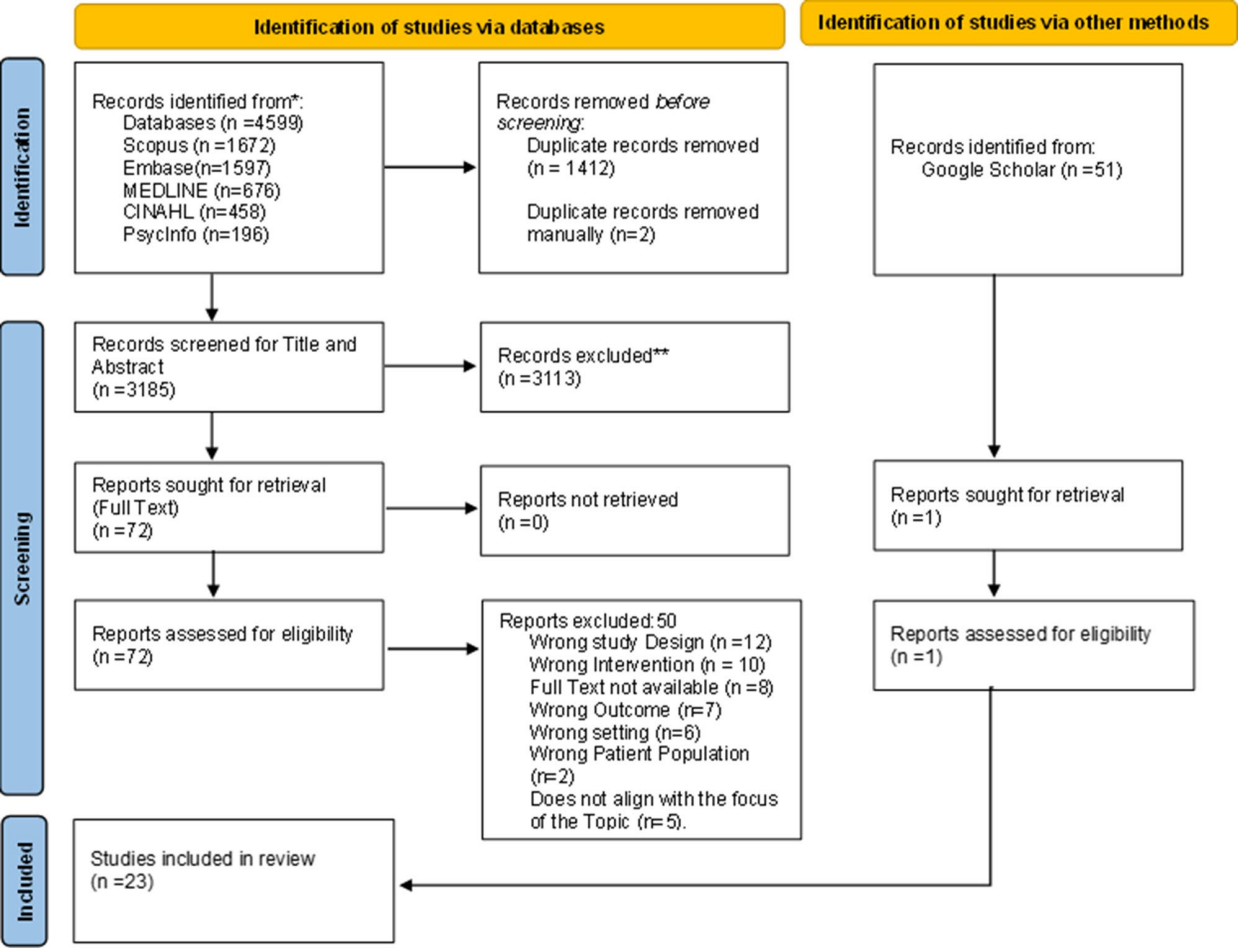
Studies included in the review were identified through a process outlined in a PRISMA 2020 [19] flow diagram

A total of 4,599 articles were imported into Covidence for screening. The review process is outlined in the PRISMA flowchart (Fig. 2). After removing 1,414 duplicates, 3,185 articles remained for title and abstract screening. Following this stage, 3,113 articles were excluded based on relevance to the research question. Subsequently, 72 full-text articles were assessed for eligibility. Of these, 50 articles were excluded following full-text review. The primary reasons for exclusion included the use of an inappropriate intervention, such as telehealth or telephone follow-up, rather than in-person NLC, and study designs that did not meet the inclusion criteria. In addition, one relevant thesis was identified through Google Scholar. Overall,23 studies were included in the final synthesis.

The studies were conducted across a wide range of countries: Nine studies were based in the United Kingdom [30–38]; six were conducted in various European countries, including Sweden, Denmark, Ireland, and the Netherlands [39–44]; five in Australia [45–49]; two in Hong Kong [50, 51] and one in West Africa [52].

Most studies were conducted in outpatient settings, with a major focus on cardiology, oncology, and rheumatology. Studies included in this review evaluated patient satisfaction and experiences with NLCs, primarily employing mixed quantitative and qualitative methodologies. In particular, 12 studies employed a cross-sectional design with structured patient satisfaction surveys, while seven studies used a qualitative approach, relying on semi-structured interviews and thematic analysis. Additionally, four studies employed mixed methods designs, combining survey data with qualitative analysis to provide a comprehensive understanding of patient experiences. The study characteristics are presented in Appendix 3. The focus of the included studies varied; however, the primary objective of these studies is to evaluate patient satisfaction, explore patient experiences and assess service accessibility with NLCs across diverse health care settings and countries.

Twenty out of twenty-three studies explicitly focused on patient satisfaction outcomes. The patient experience in the nurse-led clinic emerged as the primary theme across the included studies. The subthemes identified under this theme included patient satisfaction with the clinic, knowledge about the disease, acceptability of clinics managed by APN, and the care and support provided by the APN, which encompassed elements such as professionalism, continuity of care, and a holistic approach. The second primary theme was the impact of the nurse-led clinic on the accessibility of care, clinical outcomes, and the timeliness of care. The summary of key themes and corresponding studies, along with the quality of the studies, is presented in Table 2. The key finding mapped to outcomes are presented in a summary Table (Appendix 4).

### Study quality

#### Quality appraisal

Critical appraisal is a fundamental component of a systematic review, as it ensures the trustworthiness, methodological rigour, and relevance of the included studies. It strengthens the overall reliability and credibility of the review findings by systematically assessing the quality of the evidence, which underpins evidence-based practice [53].

In this review, the Joanna Briggs Institute (JBI) Critical Appraisal Checklist was employed to evaluate the methodological quality of the included studies. The JBI Qualitative and Analytical Cross-Sectional tools [20, 54] were selected to evaluate the studies that utilised a qualitative approach, the cross-sectional design, and the mixed method design. Each tool provides comprehensive criteria addressing research congruity, sampling methods, data collection and analysis, representation of participant perspectives, and ethical considerations. The results of the critical appraisal are presented in Appendix 2.

Two researchers independently conducted the critical appraisal of each study to minimise bias and enhance objectivity. In cases of disagreement or inconsistency in quality ratings, a third reviewer was consulted to make the final decision. This structured, collaborative process helped identify the most robust and credible evidence to address the research question, and our synthesis focused on patients’ perceptions and experiences with NLCs.

The quality appraisal in this review was used to interrogate and understand the methodological quality of the studies, rather than to exclude them. Studies were retained even when specific methodological details were limited, recognising that reporting constraints such as journal word limits may have restricted authors from providing comprehensive descriptions of their methods [55].

### Patient experiences

The quantitative and qualitative findings were integrated and presented in Table 3, allowing for comparison and contrast of the findings. The process of qualitative synthesis using the JBI approach is provided in Appendix 2, and the GRADE-CERQual summary of the qualitative findings is detailed in Appendix 5

**Table 3:** Integrated findings

Integrated Findings			
Themes	Subthemes	Qualitative findings	Quantitative Findings
**Patient Experiences**	Patient satisfaction	(1) Patients were extremely satisfied with the nurse-led clinic **(C)** [30]. (2) High satisfaction with the physical facilities and the appointment systems at the nurse-led diabetes clinic (**C)** [52].	(1) 100% of patients received answers to their questions to their satisfaction. [30]. (2) 2.98% rated their experience at the nurse-led clinic as 10 out of 10 [42]. (3) 3.78% of clients rated the overall service at the nurse-led clinic as satisfactory [50]. (4) 4.97% of patients participating in clinical trials were pleased to be reviewed in a nurse-led setting [38].
	Knowledge about the disease	(1) Nurses spent time with participants and explained the information well **(U)** [31]. (2) Increased understanding of the disease process and monitoring their health **(C)** [49]. (3) Increased knowledge about the disease and treatment **(U)** [44].	(1) 91.7% of patients stated they received appropriate support and knowledge to self-manage their side effects [41]. (2) 95.5% indicated consultation, and the findings provided an in-depth understanding of the impairment and decline in different cognitive domains [50]. (3) 75.4% of participants reported a much better understanding of their illness after seeing the specialist nurse. [36].
	Care and support	(1) Person-centred care and support, combined with streamlined care through organising services and advocating for patients’ needs, contributed to improved patient experiences **(U)** [39]. (2) Rapport and trust, along with continuity of care, are essential for a positive patient experience **(C)** [45]. (3) Participants experienced a sense of security due to the person-centred approach of nurses **(U)** [43]. (4) Patients felt that excellent care was provided and appreciated the support and professionalism in delivering services. **(C)** [48]. (5) Patient-centred care in the nurse-led Rheumatoid Arthritis clinic empowered patients to express themselves and make joint decisions with the nurse **(U)** [51]. (6) (6) Individualised care and reassurance were well received, providing a positive experience for the patient. **(C)** [35].	(1) The average patient-reported continuity of care across four measurement points was 62% [40]. (2) 97% of patients agreed that CNS had listened to their individual preferences and needs. [41]. (3) 94.2% of respondents strongly agreed that they had good continuity of care with specialist nurse-led follow-up [36].
	Acceptability	(1) Patients reported being satisfied with the care provided by CNS in the nurse-led clinic **(U)** [30]. (2) Patients expressed high satisfaction and comfort with the nurse-led care **(C)** [46]. (3) Patients reported positive experiences at the nurse-led clinic, noting that nurses were experienced, exceptionally well prepared, and well organised **(C)** [41]. (4) Participants perceived CNS offered a superior understanding of their condition and implications than doctors **(U)** [31]. (5) Participants experienced competence in the encounter with the nurse **(U)** [43]. (6) Confidence in the treatment provided by nursing professionals (**U)** [51]. (7) Competence of the nurses gave participants a sense of security (**U)** [44].	(1) 100% of patients were ‘extremely satisfied’ with the CNS [30]. (2) 71.9% of patients indicated that nurses were informed and up to date on their previous care, 83.8% stated that nurses provided clear instructions for managing their health, and 71.5% reported that nurses recommended a treatment for a particular health problem [46]. (3) 75% participants reported satisfaction with the care provided by the diabetic specialist nurse [32]. (4) 93% of participants preferred being treated by a specialist nurse over a general practitioner (GP) [33]. (5) 69.23% viewed Nurse Practitioners as specialists in aging, and the experience of the NP assessment was described positively [34]. (6) 98.6% respondents felt that the specialist nurse was knowledgeable about their case [36]. (7) 88% patients believed their needs were met in the nurse-led clinic [37]. (8) 92% respondents indicated that they were confident in being managed by a nurse [38].
**Impact of Nurse-led clinic**	Accessibility	(1) Patients knew how to contact the nurse, felt the nurse took time and was more available than physicians **(U)** [43]. (2) Devote more time to patients, as the consultation time is longer than that of a standard medical consultation with a specialist, **(C)** [51].	(1) 80% patients are satisfied with the waiting times in the nurse-led clinic [46]. (2) 90% of participants rated very good or excellent for having the colorectal nurse as a single point of contact [48].
	Clinical Outcomes		(1) HbA1c reduced by 1.14% (*p* < 0.0001); cholesterol reduced by 0.4 mmol/L (*p* < 0.0001); no significant BP change [32]. (2) There is a 0.4% mortality rate within 30 days from non-cardiac causes. The readmission rate within 30 days is 10%, with 1.8% being cardiac-related, and 1.1% requiring repeat revascularisation [42].
	Timeliness of care	(1) Satisfaction with waiting time to see the clinician **(C)** [30].	(1) 95% of patients were seen within 15 minutes of the appointment time [30]. (2) RACPC significantly improved timeliness of care, reducing referral-to-clinic intervals from 83 to 11 days and clinic-to-testing from 107 to 18 days (*p* < 0.001), with 98% of surveyed patients rating the service as good to excellent [47]. (3) 81.2% of participants felt that the time they spent with the specialist nurse was not too short [36].

#### Patient satisfaction

Five articles demonstrated a consistent sub-theme of patient satisfaction. Of these five studies, three scored 75% or higher on the quality assessment using the JBI quality appraisal tool. Findings across multiple studies demonstrate consistently high patient satisfaction with NLC services, reinforcing their role in meeting patient needs. Kor et al. [50] reported that 90% of older adults with cognitive complaints were either satisfied or very satisfied with care received through a dedicated nurse-led service. Similarly, Fishburn and Fishburn [30] found that all 36 participants in a nurse-led thyroid cancer follow-up clinic survey expressed 100% satisfaction with the specialist nurse assessments.

In a nurse-led diabetes management clinic, 98% of 134 surveyed patients were satisfied with the quality of care provided [52]. Likewise, Winter et al. [38] reported that 97% of patients participating in clinical trials were pleased to be reviewed in a nurse-led setting. One patient commented, *“The treatment and care I received was excellent”. “No criticism whatsoever from any aspect or person involved from beginning to end of my treatment”.*

Ibrahim et al. [42] evaluated a nurse-led follow-up clinic for patients post-percutaneous coronary intervention and found overwhelmingly positive outcomes. Of the 50 survey respondents, 98% rated the clinic 10/10, and the remaining 2% rated it 9/10.

These results collectively suggest that NLCs are well-received across diverse clinical settings, highlighting the capacity of nurse specialists to provide high-quality, patient-centred care and generate strong levels of satisfaction.

#### Knowledge about the disease

Knowledge gains were a key outcome across six studies. Three of these studies utilised mixed-method designs, two were qualitative, and one was cross-sectional. All of these studies demonstrated more than 75% quality in the cross-sectional studies and over 80% in the qualitative studies, as assessed by the JBI tool. Kor et al. [50] reported that 99.5% of patients obtained a deeper understanding of cognitive impairment and dementia following consultation. Repeated interactions with nurses enhanced disease-related knowledge and promoted adherence to prescribed therapies [44]. One participant stated: “… *when I had the opportunity to talk to her and pose all my questions … that is what has made me take the medications”.*

Participants described nurse-led explanations as more accessible and relatable than those from other sources. In Habibi et al. [31], a participant stated:“*I felt that the [clinical nurse specialist] had a better day-to-day understanding of the condition and implications than I have had been explained by a doctor.*” Ramachandran et al. [49] similarly found that patients valued clear explanations of cirrhosis and required tests, with one participant remarking: “*I needed a lot of information, and they explained it in layman’s terms”.*

Across Gyldenvang et al. [41] and Vanalia et al. [36], approximately 90% of participants felt adequately supported in managing side effects. Engagement with specialist nurses contributed to improved understanding of disease processes and self-management.

#### Care and support

Nine studies identified the subtheme of care and support. One study demonstrated a quality score of 37.5%, while the rest showed scores of 75% or above for cross-sectional studies and 80% or above for qualitative studies, assessed using the JBI tool. In this subtheme, care and support were defined as continuity of care, person-centred care, and a holistic approach. Bala et al. [39] noted that care was tailored to individual disease experiences, delivered with empathy and expertise. One participant reflected: *“Y*ou feel that she is interested in my problem and that she really wants to help.”

Continuity of care emerged as an important theme. Berglund et al. [40] reported that 62% of patients identified continuity as important, with 36% rating it very important and 40% quite important. Similarly, Vanalia et al. [36] found that 94.2% of participants experienced good continuity in nurse-led follow-up. Bennett-Daly et al. [45] also reported participants valued the opportunity to see the same APN consistently. One of the participants commented, *“Since I’ve been in Launceston for the last six months, I’ve been coming here to see the [nurses] every Thursday when I can”.*

Patients appreciated the private and individualised support offered by specialist nurse services. Taylor et al. [35] reported that participants felt reassured and safe during consultations: “*A safe space, ask questions and get reassurance and the right answers.”* Another participant added: “*Someone that you can speak to and address the problems that you do not get the time with the doctors to talk about.”* As also reported in Bennett-Daly et al. [45], trust in NLCs was attributed to building rapport with the participants, expressing, *“They actually listen to me and they don’t judge you”.*

Petrushnko et al. [48] found that over 90% of respondents rated their colorectal cancer nurse highly for care coordination and information provision, with 74% rating their professional conduct as excellent.

Pun et al. [51] emphasised the emotional support provided by nurse-led rheumatology clinics. One participant explained: “They would comfort me, which in turn helped with my medication use and emotional stability”. Patients consistently preferred consulting with the same nurse, highlighting the benefits of relationship continuity.

Gyldenvang et al. [41] reported that 97% of participants were satisfied with how their personal needs were addressed. Larsson et al. [43] observed that trust was cultivated when nurses treated patients as individuals and took a genuine interest in their well-being.

#### Acceptability

In this review, the acceptability of the nurse-led clinic refers to the professionalism and competence of the APN. The studies demonstrated a high level of acceptability for the APN approach in NLCs across various clinical settings. Around 70% of these studies show quality of more than 75% in both cross-sectional and qualitative in the JBI quality assessment tool. This was reflected in patients’ willingness to participate in nurse-led follow-up, their confidence in the quality of care provided, and overall satisfaction with the APN model. In the study by Pun et al. [51], participants described specialist nurses as competent and capable, stating, *“Nurses not only help other patients and me but also assist with alleviating the doctors’ workload and stress. Nurse specialists really are more than half a doctor”.* Similarly, Winter et al. [38] reported that 92% of participants attending a nurse-led thyroid clinic expressed confidence in the care provided by an APN, and only 11% preferred to be reviewed by a physician.

Several studies found overwhelmingly positive patient satisfaction levels with nurse-led care. Fishburn and Fishburn [30] reported 100% satisfaction with nurse-led thyroid cancer follow-up assessments, and Habibi et al. [31] found that 94.5% of participants were satisfied with being seen by a clinical nurse specialist. In Drewry et al. [33], 93% of participants preferred being treated by a specialist nurse over a General Practitioner (GP), while only 7% preferred GP care. Hicks et al. [32] found that 88% of 121 respondents believed diabetes specialist nurses supported them with medication review and lifestyle education, and 97% felt included in treatment decisions.

Confidence and the perceptions of confidence in APN care were consistent across multiple studies. In Gyldenvang et al. [41], participants highlighted the professionalism and preparation of nurses: “*I experienced competent and forthcoming nurses who were prepared for each consultation and familiar with my treatment plan”.* Larsson et al. [43] and Sjö and Bergsten [44] similarly reported that participants experienced a strong sense of nurse competence and appreciated the disease-specific knowledge that provided a sense of security. Many participants viewed nurse specialist care as complementary to physician-led care, especially in specialist areas such as rheumatology. One participant commented that “*both the nurse and the doctor are highly specialized, so I never think that the nurse I’m meeting will be lacking in competence”.*

Stirling et al. [34] found that 69% of participants considered nurse practitioners to be healthcare specialists who were preferred over other professionals due to their ability to take time to communicate, explain, and listen, and one participant commented, *“the doctor hasn’t got time … is a busy man.”* Similarly, Habibi et al. [31] highlighted that participants appreciated the clarity, organisation, and supportive environment provided by nurses in clinic settings.

Further reinforcing confidence in the APN model, Vanalia et al. [36] reported that 98.6% of participants believed the specialist nurse was knowledgeable about their case. In contrast, in the study by Williams et al. [37], only 7% of participants who were reviewed exclusively by nurses felt their care would have benefited from additional doctor input.

### Impact of the nurse-led clinic

#### Accessibility

Another theme that emerged is the impact of the nurse-led clinic, with a subtheme focusing on the accessibility of these clinics. Accessibility refers to participants’ ability to contact specialist nurses when needed. It is characterised by ease of access, responsiveness, and convenience. Out of the four studies that discuss about the accessibility of NLCs, only one had less than 50% quality assessment.

In the study by Petrushnko et al. [48], 86% of participants highly rated their access to colorectal cancer nurses, underscoring the importance they placed on timely and responsive specialist nursing support. Similarly, a study by Pun et al. [51] focusing on a nurse-led rheumatoid arthritis clinic reflected accessibility through the ease of booking appointments and the availability of multiple contact methods, including a dedicated nurse-led hotline. This immediate access was described by a participant as, *“when I encounter things that are unclear and don’t know who to ask, I call the nurse, for example, to inquire about the side effects of medications. I don’t have to wait long”.*

This was further supported by findings from Coleman et al. [46], where 80% of patients receiving kidney nursing services reported being seen within 30 minutes of their scheduled appointment. This level of promptness and the convenience not only contributed to patient satisfaction but also strengthened the perception that NLCs are highly accessible and tailored to patient needs.

In the study conducted by Larsson et al. [43], participants observed that nurses had more time available compared to physicians, allowing nurses to perform thorough assessments and address patient concerns with care and attention. These findings indicate that NLCs are accessible and nurture an environment where patients feel supported and receive timely professional advice.

#### Clinical outcomes

The studies included in this review provide evidence supporting the clinical effectiveness of NLCs. Hicks et al. [32] reported improved clinical outcomes in patients managed by nurse practitioners, with a mean HbA1c reduction of 1.14% and an 8.26% drop in total cholesterol over six months (*p* < 0.0001). Although blood pressure changes were not significant, metabolic markers showed positive trends. However, the quality of this study was identified as less than 50.

Ibrahim et al. [42] demonstrated the safety of the model, with only 0.4% mortality (all non-cardiac), 10% 30-day readmission (1.8% cardiac-related), and 1.1% requiring repeat revascularisation. The quality assessment score of this cross-sectional study was more than 75%. These outcomes indicate that nurse-led care is both safe and clinically effective.

#### Timeliness of care

Studies included in this review demonstrate that NLCs consistently provide timely care. Nguyen et al. [47] reported a statistically significant reduction in waiting times (*p* < 0.001). Similarly, Fishburn and Fishburn [30] found that 80–95% of participants were seen within 10 to 15 minutes, with delays mainly due to the prioritisation of newly diagnosed cancer patients. Both the above cross-sectional studies demonstrated low quality. Vanalia et al. [36] reported that 81.2% of patients felt the time spent with specialist nurses was adequate, although 14.5% desired slightly more time. This mixed-method study had a high-quality score (>75%). Overall, these findings suggest that nurse-led services generally deliver prompt care, with high level of patient satisfaction regarding the timeliness of care provided.

Overall, the findings of this study suggest that the advanced nurse practitioner model is highly acceptable to patients, with high levels of satisfaction, confidence, and perceived competence noted across a wide range of clinical settings. However, some studies did highlight drawbacks. Winter et al. [38] found negative responses about long wait times and delayed appointments, while other studies highlighted inconsistencies in information provision. Coleman et al. [46] reported inconsistent communication and provision of information about lifestyle modifications, and the need for nurses to conduct a thorough assessment prior to judging the information, education, and support needs of patients. Berglund et al. [40] found that about half the patients wished for more information to be provided. Both studies highlight the need for a patient-centred approach in the provision of information and education.

## Discussion

This mixed-methods narrative synthesis explored patient perceptions of NLCs across various settings. The identified themes showed positive findings regarding patient-reported outcomes, including satisfaction with the nurse-led clinic, knowledge about their disease, care and support, acceptability of the advanced practice nurse-led care, accessibility, timeliness of care and objectively measured clinical outcomes.

The findings from this review demonstrate that NLCs have surpassed the needs of patients, resulting in high satisfaction rates across various contexts and settings. This suggests that NLCs are perceived positively by participants, as confirmed through our qualitative findings. Across the studies, most participants were satisfied with the care provided in NLCs. This finding is consistent with previous studies [18, 56].

Studies included in this review consistently demonstrated high levels of satisfaction with NLCs. A prominent theme identified was the increase in patients’ knowledge about their disease conditions. This was largely attributed to the use of clear and detailed explanations delivered in layman’s terms, which not only improved medication adherence and confidence in self-management but also empowered patients to be more actively involved in their care. The in-depth understanding patients gained reflects the effectiveness of nurse-led consultations in translating complex clinical information into comprehensible, person-centred communication. These findings confirm the literature, which suggests that periods of emotional vulnerability, when patients may forget to ask questions or feel hesitant to voice concerns, individualised follow-up conversations that focus on specific rather than general information help patients feel more supported and confident [57]. Studies synthesised in the qualitative systematic review by Jakimowicz [14], similarly highlighted the provision of information about disease processes and care as a critical component of patient experience in nurse‑led settings and emphasised that patient education was a central aspect of care [58], which demonstrated that informed decision‑making enhanced patients’ sense of empowerment and their ability for self‑management. Furthermore, the findings align with this review [31, 36, 41, 44, 49, 50], which suggests that effective communication must be tailored to the patient’s level of understanding.

The approach taken by nurses, characterised by empathy, active listening, and relational engagement, plays a crucial role in building therapeutic relationships and fostering trust among patients. This is especially significant given the specialist knowledge that nurses possess, which often complements that of doctors and contributes to patients’ sense of security, as highlighted in these studies [43, 44, 51].

Effective communication is a cornerstone of these therapeutic relationships, as it supports shared decision-making and enhances patient self-management [59]. By tailoring their explanations to meet individual needs, nurse-led consultations help bridge knowledge gaps, particularly for individuals from culturally and linguistically diverse backgrounds. While the overall feedback was highly positive, a small number of participants expressed the need for additional information or longer consultation times, underscoring the importance of allocating adequate time and maintaining ongoing engagement with patient needs. This review highlights that the communication style and familial approach used in NLCs are key facilitators of patients’ increased knowledge, confidence, and engagement in care. A similar systematic review of qualitative studies on nurse-led care in rheumatoid arthritis also resonates with this study’s findings on increasing patient knowledge [60]. These findings not only consolidate the value of nurse-led models but also underscore the critical role of communication in delivering safe, person-centred healthcare.

The holistic and person-centred nature of nurse-led care was evident in the studies reviewed. One significant aspect of person-centred care that participants valued was continuity. Participants expressed significant appreciation for the continuity of care provided by the NLCs. Qualitative findings on care and support, particularly continuity of care, were consistent with the quantitative evidence, as presented in the results section and summarised in the Integrated Findings Table 3. This finding aligns with the quantitative systematic review conducted by Davis et al. [61], which examined the effectiveness of nurse-led services for individuals with chronic diseases. Their study found that continuity of care significantly contributed to better patient retention in disease management programs.

Additionally, a systematic review by Garner et al. [62] on the quality of care in nurse-led services for patients with rheumatoid arthritis reported a positive perception of continuity of care. Interestingly, one study revealed a divergence between quantitative and qualitative findings regarding continuity of care. Besides continuity, the holistic nature of nurse-led care was a recurring theme, with participants frequently noting that they felt listened to and received individualised care. This approach facilitated trust and the development of a therapeutic relationship. Garner et al. [62] also highlighted the holistic focus of nurse-led services in rheumatoid arthritis care, which aligns with the findings of our review. These observations are further supported by previous qualitative evidence synthesised by Jakimowicz [14], in which two included studies identified the holistic approach adopted by nurses as a key contributor to positive patient experiences.

The findings of this review are congruent with those of Htay and Whitehead [63], who found that ANP-led care is non-inferior to physician-led or usual care in terms of clinical effectiveness. Garner et al. [62] also reported high patient acceptability of nurse-led care in the management of rheumatoid arthritis. Patients’ confidence and level of engagement were shown to rely heavily on the specialist nurses’ clinical competence and medical expertise, as further evidenced by Jakimowicz [14].

The systematic review by Woo, Lee, and Tam [64] focused on nurse practitioner-led care in emergency and critical care settings and reported shorter time to treatment in both settings. Although this outcome reflects clinical efficiency rather than patient perceptions, it supports the positive patient perception of care timeliness that emerged in our review.

Along with other literature on NLCs, which emphasised their accessibility, our review also identified patient satisfaction with accessibility, characterised by reduced waiting times, ease of contact, responsiveness, and timely access to treatment. This aligns with findings from Randall et al. [18], who reported that approximately one-third of included studies highlighted that accessibility to care is the key benefit of nurse-led care. Although Connolly and Cotter [2] noted the effectiveness of NLCs in improving access to healthcare services in their umbrella review, they also asked for further research to explore this aspect in greater depth. Only 9% of studies in this systematic review measured the objective clinical outcomes of NLCs and reported positive outcomes, demonstrating that nurse-led care is both safe and clinically effective. This review also identified that there are studies investigating the clinical outcomes of nurse-led care, except for those in emergency and critical care settings.

Although this review found that overall satisfaction with NLC was high, with the highest satisfaction reported in representing patients [46], another study [52] showed higher participation rates among female patients. There were, however, some drawbacks, with a few studies indicating negative responses from patients, such as their preference to see a consultant or GP [36–38] and suggestions that communication could be improved between the NLC and GP [33]. In addition, we noted greater emphasis on the evaluation of patients’ subjective experiences, with very few studies examining clinical outcomes from the NLC model of care. This indicates a research gap and highlights the need for future studies investigating objective health indicators in NLCs. Further studies on the clinical outcomes of NLCs would help provide a fuller picture of not only acceptability and patient satisfaction but also the clinical implications of this model of care.

As seen in our integrated findings, several qualitative findings explain and support the quantitative findings. However, as some results were incongruent, further mixed-methods studies are warranted that integrate quantitative and qualitative data.

## Limitations

Our systematic review has several limitations. Some studies had small sample sizes, and some of the larger studies identified disparity amongst genders, disease types, and ages. For instance, Gyldenvang et al. [41] were only investigating female patients with breast and gynaecological cancers, so it does not provide insight into male patient perspectives or women with other cancers. Berglund et al. [40] had a large sample size but had a statistically significantly lower response rate for younger patients and patients with head and neck, skin, lung, and brain cancers. This heterogeneity among the included studies may make generalising findings across different service types, ages, and genders difficult.

Likewise, a limitation of the approach of this systematic review may be that by not including NLCs in the fields of paediatric and child and family health, maternity services and drug, sexual and mental health services, our findings on patient acceptability and continuity of care may not be able to be generalised across a variety of models of care. Although the decision to include studies published from 2012 onwards was made to build on the most recent systematic review, this restriction may have resulted in the exclusion of earlier foundational work that could have provided additional historical context. While this approach ensured a focus on contemporary literature, it may have excluded earlier influential studies and introduced a degree of temporal bias.

The exclusion of patient experience of nurse-led telehealth clinics may also have implications, especially given the expansions of virtual health and telehealth services post-COVID. Our review was limited to studies published in the English language only due to a lack of resources for translation, which may impact the comprehensiveness of the search. Despite our best efforts to conduct a systematic search using a rigorous approach across several electronic databases, we cannot exclude the possibility of missing studies.

## Conclusion

This systematic review provides a comprehensive synthesis of patient perceptions of NLCs across various healthcare settings. It is unique in that it is the first to draw on mixed-methods studies. The findings consistently indicate high levels of patient satisfaction, demonstrating that the acceptability of advanced nurse practitioner-led care, improved patients’ knowledge, and confidence in managing their disease. As well as the positive experience of the accessibility and timeliness of care and continuity of care. These findings reflect the strengths of NLCs in delivering holistic, person-centred care supported by effective communication and advanced clinical expertise.

While the review reinforces the acceptability and perceived value of nurse-led models, the evidence primarily focuses on patient experiences in these clinics. While studies outside the scope of this review have addressed the clinical effectiveness of NLCs, they further strengthen the case for this care model. Overall, this review confirms the significant contribution of NLCs to patient-centred healthcare and underscores the need for a broader evidence base to support their ongoing development and integration into mainstream service delivery.

## Electronic supplementary material

Below is the link to the electronic supplementary material.

Supplementary material 1

Supplementary material 2

Supplementary material 3

Supplementary material 4

Supplementary material 5

## Abbreviations

NLC: Nurse Led Clinic
APN: Advanced Practice Nurses
JBI: Joanna Briggs Institute
PRISMA: Preferred Reporting Items for Systematic Reviews and Meta-Analyses
GP: General Practitioner

## Declarations

### Data Availability

No datasets were generated or analysed during the current study.
