# Supplementary material for: Perceptions, perspectives and experiences of adult patients attending nurse-led clinics: a mixed-method systematic review
Source: BMC Nurs. 2026 May 20;25:620. doi: 10.1186/s12912-026-04740-7 (PMC13366822; doi:10.1186/s12912-026-04740-7)
Supplement: Supplementary file 1 — Supplementary material 1 [file 12912_2026_4740_MOESM1_ESM.docx]

**Appendix 1: Search History**

[Run Search](https://access.ovid.com/custom/redirector/ciap.html?dest=https://acs.hcn.com.au/?acc=36422&url=https://ovidsp.ovid.com/ovidweb.cgi?T=JS&NEWS=N&PAGE=main&SHAREDSEARCHID=4yoJBTigAu4QpusT6Isx5YA3wxrXGzWVLOOz4mOztkISv2rLMnFdyYlYw4jXRN33i)

**Ovid MEDLINE**® including Daily update <1996-current>

| **#** | **Query** | **Results from 16 Apr 2025** |
| --- | --- | --- |
| 1 | "patient*".ab,ti. | 6,193,899 |
| 2 | "Client*".ab,ti. | 54,574 |
| 3 | "consumer*".ab,ti. | 78,327 |
| 4 | 1 or 2 or 3 | 6,303,180 |
| 5 | "Nurse-led clinic*".ab,ti. | 392 |
| 6 | "Nurse managed center*".ab,ti. | 53 |
| 7 | Practice Patterns, Nurses'/ | 3,029 |
| 8 | nurse-led.ab,ti. | 4,956 |
| 9 | 5 or 6 or 7 or 8 | 7,550 |
| 10 | "attitude*".ab,ti. | 145,796 |
| 11 | "Perce*".ab,ti. | 1,138,139 |
| 12 | "Satisfaction*".ab,ti. | 157,595 |
| 13 | "Patient perception*".ab,ti. | 4,380 |
| 14 | "patient experience*".ab,ti. | 20,879 |
| 15 | "Patient satis*".ab,ti. | 40,579 |
| 16 | "Patient preference*".ab,ti. | 9,785 |
| 17 | "Patient opinion*".ab,ti. | 398 |
| 18 | "Patient attitude*".ab,ti. | 969 |
| 19 | "Patient feeling*".ab,ti. | 83 |
| 20 | "Patient view*".ab,ti. | 620 |
| 21 | Patient-centred care.ab,ti. | 2,356 |
| 22 | Patient Satisfaction/ | 89,656 |
| 23 | Patient Preference/ | 11,906 |
| 24 | Patient-Centered Care/ | 24,201 |
| 25 | Nurse-Patient Relations/ | 23,558 |
| 26 | "Nurse patient relationship*".ab,ti. | 820 |
| 27 | 10 or 11 or 12 or 13 or 14 or 15 or 16 or 17 or 18 or 19 or 20 or 21 or 22 or 23 or 24 or 25 or 26 | 1,455,679 |
| 28 | 4 and 9 and 27 | 1,701 |
| 29 | limit 28 to (english language and yr="2012 -Current" and ("young adult (19 to 24 years)" or "adult (19 to 44 years)" or "young adult and adult (19-24 and 19-44)" or "middle age (45 to 64 years)" or "middle aged (45 plus years)" or "all aged (65 and over)" or "aged (80 and over)")) | 677 |

**Embase** <1996 to 2025 April 14>

[Run search](https://access.ovid.com/custom/redirector/ciap.html?dest=https://acs.hcn.com.au/?acc=36422&url=https://ovidsp.ovid.com/ovidweb.cgi?T=JS&NEWS=N&PAGE=main&SHAREDSEARCHID=4dhitoNFd8AdwI1IeqBuH5qrS37lChY5Nvekp9Y33VFeE7o9hhR2VMgLZF3KB6GhO)

| **#** | **Query** | **Results from 16 Apr 2025** |
| --- | --- | --- |
| 1 | "patient*".ab,ti. | 11,558,364 |
| 2 | "Client*".ab,ti. | 81,356 |
| 3 | "Consumers*".ab,ti. | 65,066 |
| 4 | 1 or 2 or 3 | 11,675,374 |
| 5 | "nurse led clinic*".ab,ti. | 1,088 |
| 6 | "nurse managed center*".ab,ti. | 56 |
| 7 | nurse-led.ab,ti. | 9,658 |
| 8 | 5 or 6 or 7 | 9,714 |
| 9 | "attitude*".ab,ti. | 229,200 |
| 10 | "Perce*".ab,ti. | 1,892,886 |
| 11 | "Satisfaction*".ab,ti. | 272,167 |
| 12 | "patient perception*".ab,ti. | 8,488 |
| 13 | "patient experience*".ab,ti. | 47,390 |
| 14 | "patient satis*".ab,ti. | 74,131 |
| 15 | "patient preference*".ab,ti. | 19,537 |
| 16 | "patient opinion*".ab,ti. | 834 |
| 17 | "patient attitude*".ab,ti. | 1,835 |
| 18 | "patient feeling*".ab,ti. | 216 |
| 19 | "patient view*".ab,ti. | 1,223 |
| 20 | "patient centred care*".ab,ti. | 4,016 |
| 21 | exp Client Satisfaction/ | 0 |
| 22 | exp Client Attitudes/ | 0 |
| 23 | exp Patient Centered Care/ | 3,325 |
| 24 | nurse-patient relationship.mp. | 23,860 |
| 25 | 9 or 10 or 11 or 12 or 13 or 14 or 15 or 16 or 17 or 18 or 19 or 20 or 21 or 22 or 23 or 24 | 2,329,965 |
| 26 | 4 and 8 and 25 | 2,320 |
| 27 | limit 26 to (english language and ("300 adulthood (age 18 yrs and older)" or "320 young adulthood (age 18 to 29 yrs)" or "340 thirties (age 30 to 39 yrs)" or "360 middle age (age 40 to 64 yrs)" or "380 aged (age 65 yrs and older)" or "390 very old (age 85 yrs and older)") and yr="2012 -Current") [Limit not valid in Embase; records were retained] | 1,891 |
| 28 | "Patient*".ab,ti. | 11,558,364 |
| 29 | "Client*".ab,ti. | 81,356 |
| 30 | "Consumers*".ab,ti. | 65,066 |
| 31 | 28 or 29 or 30 | 11,675,374 |
| 32 | "nurse-led clinic*".ab,ti. | 1,088 |
| 33 | "nurse managed center*".ab,ti. | 56 |
| 34 | nurse-led.ab,ti. | 9,658 |
| 35 | nursing practice/ | 6,730 |
| 36 | 32 or 33 or 34 or 35 | 16,049 |
| 37 | "Perce*".ab,ti. | 1,892,886 |
| 38 | "Attitude*".ab,ti. | 229,200 |
| 39 | "Satisfaction*".ab,ti. | 272,167 |
| 40 | "Patient perception*".ab,ti. | 8,488 |
| 41 | "patient experience*".ab,ti. | 47,390 |
| 42 | "patient satis*".ab,ti. | 74,131 |
| 43 | "patient preference*".ab,ti. | 19,537 |
| 44 | "patient opinion*".ab,ti. | 834 |
| 45 | "patient attitude*".ab,ti. | 1,835 |
| 46 | "patient feeling*".ab,ti. | 216 |
| 47 | "patient view*".ab,ti. | 1,223 |
| 48 | patient centred care.ab,ti. | 4,006 |
| 49 | patient satisfaction/ | 181,558 |
| 50 | patient preference/ | 30,558 |
| 51 | patient attitude/ | 74,719 |
| 52 | person centered care/ | 3,325 |
| 53 | nurse patient relationship/ | 23,591 |
| 54 | 37 or 38 or 39 or 40 or 41 or 42 or 43 or 44 or 45 or 46 or 47 or 48 or 49 or 50 or 51 or 52 | 2,431,319 |
| 55 | 31 and 36 and 54 | 3,367 |
| 56 | limit 55 to (english language and yr="2012 -Current" and (adult <18 to 64 years> or aged <65+ years>)) | 1,594 |

**APA PsycInfo**

<2002 to April 2025 Week 1>

[Run Search](https://access.ovid.com/custom/redirector/ciap.html?dest=https://acs.hcn.com.au/?acc=36422&url=https://ovidsp.ovid.com/ovidweb.cgi?T=JS&NEWS=N&PAGE=main&SHAREDSEARCHID=6QBANwkj9AeyUjTSoigbOR5mVQtayrjHEWigA2nXrtoJnaV6Pxcuo6Mp50Zp3NOF0)

| **#** | **Query** | **Results from 16 Apr 2025** |
| --- | --- | --- |
| 1 | "patient*".ab,ti. | 614,347 |
| 2 | "Client*".ab,ti. | 78,001 |
| 3 | "Consumers*".ab,ti. | 41,526 |
| 4 | 1 or 2 or 3 | 723,251 |
| 5 | "nurse led clinic*".ab,ti. | 63 |
| 6 | "nurse managed center*".ab,ti. | 12 |
| 7 | nurse-led.ab,ti. | 1,267 |
| 8 | 5 or 6 or 7 | 1,279 |
| 9 | "attitude*".ab,ti. | 155,170 |
| 10 | "Perce*".ab,ti. | 567,450 |
| 11 | "Satisfaction*".ab,ti. | 103,576 |
| 12 | "patient perception*".ab,ti. | 1,114 |
| 13 | "patient experience*".ab,ti. | 3,325 |
| 14 | "patient satis*".ab,ti. | 4,417 |
| 15 | "patient preference*".ab,ti. | 1,929 |
| 16 | "patient opinion*".ab,ti. | 74 |
| 17 | "patient attitude*".ab,ti. | 289 |
| 18 | "patient feeling*".ab,ti. | 41 |
| 19 | "patient view*".ab,ti. | 205 |
| 20 | "patient centred care*".ab,ti. | 692 |
| 21 | exp Client Satisfaction/ | 5,747 |
| 22 | exp Client Attitudes/ | 20,358 |
| 23 | exp Patient Centered Care/ | 1,122 |
| 24 | nurse-patient relationship.mp. | 440 |
| 25 | 9 or 10 or 11 or 12 or 13 or 14 or 15 or 16 or 17 or 18 or 19 or 20 or 21 or 22 or 23 or 24 | 753,561 |
| 26 | 4 and 8 and 25 | 316 |
| 27 | limit 26 to (english language and ("300 adulthood (age 18 yrs and older)" or "320 young adulthood (age 18 to 29 yrs)" or "340 thirties (age 30 to 39 yrs)" or "360 middle age (age 40 to 64 yrs)" or "380 aged (age 65 yrs and older)" or "390 very old (age 85 yrs and older)") and yr="2012 -Current") | 196 |

|  | **Run date** | **14/04/2025** | **Number of results-1672** |
| --- | --- | --- | --- |
|  |  |  |  |
|  | **Concept 1** | **Concept 2** | **Concept 3** |
| **Title ABS- Key** | Patient* | Nurse -led | attitudes* |
|  | Client* | nurse led clinic* | Perce* |
|  | Consumer* | nurse managed centre | Satisfaction* |
|  |  |  | Patient perception* |
|  |  |  | Patient experience* |
|  |  |  | Patent satis* |
|  |  |  | Patient preference* |
|  |  |  | Patient opinion* |
| **Filter** |  |  | Patient attitude* |
| 2012-2025 |  |  | Patient feeling* |
| English Only |  |  | Patient view* |
| Articles only |  |  | Patient centred-care |
|  |  |  | Nurse patient relations ship |

**Scopus**

**Search string**

((( TITLE-ABS-KEY ( patient* ) ) OR ( TITLE-ABS-KEY ( client* ) ) OR ( TITLE-ABS-KEY ( consumer* ) )) AND (( TITLE-ABS-KEY ( ""Nurse led clinic*"" ) ) OR ( TITLE-ABS-KEY ( ""Nurse managed centre"" ) ) OR ( TITLE-ABS-KEY ( ""Nurse-led"" ) )) AND (( TITLE-ABS-KEY ( attitude* ) ) OR ( TITLE-ABS-KEY ( perce* ) ) OR ( TITLE-ABS-KEY ( satisfaction* ) ) OR ( TITLE-ABS-KEY ( ""patient perception*"" ) ) OR ( TITLE-ABS-KEY ( ""patient experience*"" ) ) OR ( TITLE-ABS-KEY ( ""patient satis*"" ) ) OR ( TITLE-ABS-KEY ( ""patient preference*"" ) ) OR ( TITLE-ABS-KEY ( ""patient opinion*"" ) ) OR ( TITLE-ABS-KEY ( ""patient attitude*"" ) ) OR ( TITLE-ABS-KEY ( ""patient feeling*"" ) ) OR ( TITLE-ABS-KEY ( ""patient view*"" ) ) OR ( TITLE-ABS-KEY ( ""patient centred care"" ) ) OR ( TITLE-ABS-KEY ( "" nurse patient relationship"" ) )) AND PUBYEAR > 2011 AND PUBYEAR < 2026 AND ( LIMIT-TO ( LANGUAGE,""English"" ) ) AND ( LIMIT-TO ( DOCTYPE,""ar"" ) ) )

**CINAHL**

|  | **Run date-17/03/2025** |  | **Number of results-458** |
| --- | --- | --- | --- |
|  |  |  |  |
|  | Concept 1 | Concept 2 | Concept 3 |
| **All Text Field** | Patient | nurse-led clinic | Attitude* |
|  | Client | nurse-led | Perce* |
|  | Consumer |  | Satisfaction |
|  |  |  | Patient perceptions |
|  |  |  | Patient experience |
|  |  |  | Patient satis* |
|  |  |  | Patient Preference* |
|  |  |  | Patient opinion* |
|  |  |  | Patient attitude* |
|  |  |  | Patient Feeling* |
|  |  |  | Patient View |
|  |  |  | Patient centred care |
|  |  |  | Nurse patient relationship |
|  |  |  |  |
| **Indexed Subject Headings** |  | MH "Nurse-Managed Centers" | MH "Patient Satisfaction" |
|  |  |  | MH "Patient Preference" |
|  |  |  | MH "Patient Attitudes" |
| **Limits** |  |  | MH "Nurse-Patient Relations" |
| 01/01/2012-31/12/2025 |  |  | MH "Patient Centered Care" |
| Full text |  |  |  |
| Adult aged>18 |  |  |  |
| English |  |  |  |

**Search String**

((XB patient*) OR (XB Client*) OR (XB Consumer*)) AND ((XB Nurse led clinic*) OR (XB Nurse managed center*) OR (XB Nurse-led) OR ((MH "Nurse-Managed Centers"))) AND ((XB Attitude*) OR (XB Perce*) OR (XB Satisfaction*) OR (XB Patient perception*) OR (XB Patient experience*) OR (XB Patient satis*) OR (XB Patient preference*) OR (XB Patient opinion*) OR (XB Patient attitude*) OR (XB Patient feeling*) OR (XB Patient view*) OR (XB Patient centred care) OR (XB Nurse patient relationship) OR ((MH "Patient Satisfaction+")) OR ((MH "Patient Preference")) OR ((MH "Patient Centered Care")) OR ((MH "Nurse-Patient Relations")) OR ((MH "Patient Attitudes")))
