# Supplementary material for: Perceptions, perspectives and experiences of adult patients attending nurse-led clinics: a mixed-method systematic review
Source: BMC Nurs. 2026 May 20;25:620. doi: 10.1186/s12912-026-04740-7 (PMC13366822; doi:10.1186/s12912-026-04740-7)
Supplement: Supplementary file 2 — Supplementary material 2 [file 12912_2026_4740_MOESM2_ESM.docx]

**Appendix 2: Critical appraisal results**

**Table:   Critical appraisal using the Analytical cross-sectional tool**

| **Citation** | **Q1** | **Q2** | **Q3** | **Q4** | **Q5** | **Q6** | **Q7** | **Q8** | **%** |
| --- | --- | --- | --- | --- | --- | --- | --- | --- | --- |
| Bennett – Daly et al. 2021 | Y | Y | Y | Y | N | N | Y | Y | 75% |
| Berglund et al. 2015 | Y | Y | Y | Y | Y | Y | Y | Y | 100% |
| Coleman t al 2017 | Y | Y | Y | Y | N | Y | Y | Y | 87.5% |
| Drewery et al.2012 | N | Y | N | N | N | N | N | N | 12.5% |
| Fishburn and Fishburn, 2021 | Y | Y | U | U | N | N | U | N | 25% |
| Gyldenvang et al 2022 | Y | Y | Y | Y | N | N | Y | Y | 75% |
| Habibi et al, 2023 | Y | Y | Y | Y | N | N | Y | Y | 75% |
| Hicks et al. 2012 | N | Y | Y | Y | N | N | N | N | 37.5% |
| Ibrahim et al.2019 | N | Y | N | Y | Y | Y | Y | Y | 75% |
| Kor et al. 2022 | Y | Y | Y | Y | N | N | Y | Y | 75% |
| Momoh et al. 2024 | Y | Y | Y | Y | Y | N | Y | Y | 87.5% |
| Nguyen et al.2022 | N | Y | Y | U | N | N | U | U | 25% |
| Petrushunko et al.2024 | Y | Y | U | Y | U | N | N | U | 37.5% |
| Vanalia et al.2023 | Y | Y | Y | Y | N | N | Y | Y | 75% |
| Williams et al. 2012 | Y | Y | Y | Y | N | N | Y | Y | 75% |
| Winter et al.  2012 | Y | Y | Y | N | N | N | U | N | 37.5% |

**Qualitative tool**

| **Citations** | **Q1** | **Q2** | **Q3** | **Q4** | **Q5** | **Q6** | **Q7** | **Q8** | **Q9** | **Q10** | **%** |
| --- | --- | --- | --- | --- | --- | --- | --- | --- | --- | --- | --- |
| Bala et al 2012 | Y | Y | Y | Y | Y | N | N | Y | Y | Y | 80% |
| Bennett – Daly et al. 2021 | Y | Y | Y | Y | Y | N | Y | Y | Y | Y | 90% |
| Gyldenvang et al 2022 | Y | Y | Y | Y | Y | Y | N | Y | Y | Y | 90% |
| Habibi et al. 2023 | Y | Y | Y | Y | Y | N | N | Y | Y | Y | 80% |
| Larsson et al 2012 | Y | Y | Y | Y | Y | N | N | Y | Y | Y | 80% |
| Pun et al 2023 | Y | Y | Y | Y | Y | N | N | Y | Y | Y | 80% |
| Ramachandran et al. 2022 | Y | Y | Y | Y | Y | N | N | Y | Y | Y | 80% |
| Sjo and Bergsten 2018 | Y | Y | Y | Y | Y | Y | Y | Y | Y | Y | 100% |
| Stirling et al 2016 | Y | Y | Y | Y | Y | N | N | Y | Y | Y | 80% |
| Taylor 2018 | Y | Y | Y | Y | Y | N | N | Y | Y | Y | 80% |
| Vanalia 2023 | Y | Y | Y | Y | Y | N | N | Y | Y | Y | 80% |
