# Supplementary material for: Perceptions, perspectives and experiences of adult patients attending nurse-led clinics: a mixed-method systematic review
Source: BMC Nurs. 2026 May 20;25:620. doi: 10.1186/s12912-026-04740-7 (PMC13366822; doi:10.1186/s12912-026-04740-7)
Supplement: Supplementary file 3 — Supplementary material 3 [file 12912_2026_4740_MOESM3_ESM.docx]

**Appendix 3: Study Characteristics Table**

**Quantitative**

| **Author (Year)** | **Country** | **Study Design** | **Study setting** | **Inclusion criteria** | **Number of Participants (Women)** | **Age, mean/ range (years)** |
| --- | --- | --- | --- | --- | --- | --- |
| Berglund et al.2015 | Sweden | Patient satisfaction questionnaires | Nurse-led clinic the Department of Oncology, Karolinska University Hospital, Solna | Patients attending the Nurse-led clinic in the Department of Oncology, Karolinska University Hospital, Solna | 962 (207) | 18-≥80 years |
| Coleman et al. 2017 | Australia | Cross-sectional survey | 5 CKD nurse-led clinics across Metropolitan regional and remote hospitals in Queensland | Adult patients with CKD (Non dialysis) who attended the clinic d | 311(250) | 16-91+ |
| Drewery et al.2012 | England | analytical cross-sectional survey |  | patients who attended the nurse-led heart failure clinics | 192 |  |
| Fishburn and Fishburn 2021 | The United Kingdom | Cross-sectional design, survey | Clinic at Royal Preston Hospital | Patients attending the clinic over 6 weeks spring of 2019 | 36 (predominantly female) | Under the age of 50 |
| Hicks et al. 2012 | UK | retrospective clinical audits | 9 intermediate-level diabetes centres across England | Patients attending the diabetes specialist nurse (DSN) led clinic at the nine intermediate-level diabetes centres. | 424 | 21 - 90 |
| Ibrahim et al. 2019 | Ireland | Retrospective observational study | Nurse-led PCI clinic at the University Hospital Limerick | Patient attended the nurse-led clinic 6 weeks post-PCI | 1037(288) | Mean 64.8+- 29.3sd |
| Kor et al.2022 | Hong Kong/ China | Cross-sectional design, retrospective chart review | A Nurse-led clinic operated by the Nursing Department of a university in Hong Kong | All clients who visited the clinic during January 2012 and January 2018 | 98(100) |  |
| Momoh et al.2024 | Sierra Leone | Cross-sectional survey | Bo Government Hospital (BGH) nurse-led diabetes clinic | Adults who had visited the BGH Diabetes clinic. | 134 (110) | 18-60 |
| Nguyen et al. 2022 | Australia | Cross-sectional design, patient satisfaction survey | Rapid Access Chest Pain Clinic (RACPC) at Royal Perth Hospital | Patients with new onset chest pain attended RACPC | 1542 |  |
| Williams et al. 2012 | The United Kingdom | Prospective cross-sectional study | Thoracic nurse-led clinic at St James University Hospital | Patients who attended the thoracic nurse-led clinic | 85 |  |
| Winter et al.2012 | The United Kingdom | Cross-sectional design | Gastrointestinal cancer clinical trial (GICT) clinic at one hospital | Diagnosed with a GI tumour, treated in an RCT | 42 (10) | 25% <59 years, 43% 60–69 years, 29% >70 years |

**Mixed Method and Qualitative**

| **Author** | **Country** | **Study Design** | **Inclusion criteria** | **Number of participants (Women)** | **Age, Mean/ range (Years)** |
| --- | --- | --- | --- | --- | --- |
| Bala et al.2021 | Sweden | Descriptive,  explorative design with a qualitative approach inspired  by thematic content analysis | adults with RA who had had  at least three documented contacts (visit or by tele  phone)  with any of the nurse-led clinics, of which at  least one had been a visit. | 18 (1) | 41-72 |
| Bennet-Daly et al.2021 | Australia | Mixed method study, cross-sectional, retrospective chart review, interviews | 18 years and older, had access to at least one Mission Health Nurse-led clinic (MHNC) | 174 | Mean age 42 |
| Gyldenvang et al.2022 | Denmark | A sequential multi-method approach, Surveys and focus group interviews. | Women with cervical, vulvar, or vaginal cancer, who had at least four nurse-led consultations while undergoing curative chemotherapy and radiation, as well as women with breast cancer who attended at least one nurse-led consultation during adjuvant endocrine therapy follow-up. All patients were ≥18 years and able to speak and understand Danish. | 109 | >18 yrs |
| Habibi et al.2023 | UK | Cross-sectional survey | Patients with moderate or severe congenital heart disease, classified under New York Heart Association functional classes I–IV, were initially reviewed by consultants in the adult congenital heart disease clinic. | 51(40) | Mean- 31.8, range 16-74 |
| Petrushnko et al.2024 | Australia | Mixed-method Patient satisfaction surveys | Patients 3-month post operatively who had undergone elective resection for CRC in the years 2009,2013,2016, 2018,2020 | 285 |  |
| Pun et al.2023 | Hong Kong | Purposive sample, one on one face to face interviews by a Rheumatology nurse specialist | Patients with RA more than 18 years old; proficient in spoken  Cantonese; residents of Hong Kong; and capable of  understanding and responding to a written questionnaire  and/or oral questioning | 12 (10) | 54 ±13.4 |
| Ramachandran et al.2021 | Australia | Descriptive qualitative study using semi-structured interviews | Patients who visited the nurse-led hepatology clinic at least twice and the hepatologists' clinic beforehand... | 8 (2) | 64 |
| Sjo and Bergsten 2018 | Sweden | semi-structured interviews | A strategic selection of patients who were in the intervention group in the previous RCT for rheumatoid arthritis were invited. | 15(14) | 21-79 |
| Stirling et al.2016 | Australia | Qualitative study, semi-structured interviews | Attended a once weekly nurse-led memory clinic (NMLC) in the previous 12 months | 13 |  |
| Taylor et al.2018 | Australia | Exploratory, qualitative, descriptive study | Only participants who had completed all aspects of the NLSC intervention were approached by the survivorship cancer nurse conducting the clinic intervention. Each participant  was nine months post-treatment completion and the sample  reflected an equal gender distribution and range of ages. | 10 (5) | 24-74 |
| Vanalia et al.2023 | The United Kingdom | Mixed methods | Patients who had been followed up in the nurse-led renal cancer clinic between January 2020 and May 2021. Only patients who had had a partial or radical nephrectomy were deemed eligible. | 69 |  |
