# Supplementary material for: Perceptions, perspectives and experiences of adult patients attending nurse-led clinics: a mixed-method systematic review
Source: BMC Nurs. 2026 May 20;25:620. doi: 10.1186/s12912-026-04740-7 (PMC13366822; doi:10.1186/s12912-026-04740-7)
Supplement: Supplementary file 4 — Supplementary material 4 [file 12912_2026_4740_MOESM4_ESM.docx]

**Appendix 4: Summary Table**

**Quantitative**

| **Author** | **Focus of study** | **Main outcome** | **Secondary outcome/s** | **Themes** | **Key Findings** |
| --- | --- | --- | --- | --- | --- |
| **Berglund et al.2015** | To assess patient satisfaction with the clinic’s ability to manage their postoperative needs. | The study aimed to assess patient satisfaction with the clinic’s ability to manage their postoperative needs | Assessing patient satisfaction and belief if their care would have benefited from seeing a doctor | **Care and Support** | Patient satisfaction with nurse-led clinics remained stable over time, except for continuity of care and information. About half of the patients wanted more information, and their satisfaction with it was below 70 on a 0 to 100 scale, indicating a need for improvement. No statistically significant associations were found between “satisfaction with information” and patient-reported continuity of care. A more person-centred approach, e.g., an increase of information exchange, is needed to meet the wide variety of patients' information needs and preferences |
| **Coleman et al. 2017** | Patients' satisfaction with nurse-led clinics over time, Patients' perception of received information, Associations between continuity of care and satisfaction with information. The agreement between patient reported and registered | Patient perceptions of nurse led clinics was stable over time. Satisfaction generally high, | Deficiencies were reported in information and continuity of care | **Acceptability and accessibility** | Patients were highly satisfied with the nurse-led CKD clinics, with 83.8% rating the quality of care positively and 89.4% indicating that the nurse had a positive impact on their wellbeing. However, there were gaps in lifestyle interventions, with fewer discussions about smoking, alcohol, weight loss, physical activity, nutrition, and emotions. |
| **Drewery et al.2012** | Evaluate patient's experiences of the service provided by heart failure clinics | Patients experience of the nurse led clinic to improve the service. |  | **Acceptability** | patients’ perception of the care and support they have received (90%), that the team members were courteous and professional (97%), that the patients were given sufficient time during visits (87%) and that they were given a contact number for the team (84%). The latter gave access to the team and helped them to cope with their illness (90%) |
| **Fishburn and Fishburn 2021** | Assess patient satisfaction | Report on patient satisfaction of a nurse led thyroid cancer clinic |  | **Satisfaction with the nurse-led** **clinic, Acceptability, Timeliness of care** | 100% patients would prefer face to face follow up. Patients extremely satisfied with care from CNS. 100% patients satisfied with length of consultation. 52% would likely attend a support group. |
| **Hicks et al. 2012** | To assess clinical effectiveness and patient satisfaction in consultant nurse led intermediate care services | Clinical Outcomes measured by change in HbA1c, total cholesterol, BP and patient satisfaction outcome | Patient satisfaction is the secondary outcome | **Acceptability, Clinical Outcomes** | Analysis of patient satisfaction data showed participants doubled their self-rating for diabetes self-management after attending the clinics. Those who rated their management poorly reported a sixfold improvement after the intervention. The majority (97%) felt included in treatment decisions, 80% made changes to their diabetes management, and valued medicines review and lifestyle advice by the CNS. "Clinical Effectiveness: HbA1c dropped by 1.14% (from 9.53% to 8.39%, p < 0.0001). Cholesterol decreased by 0.4 mmol/L (p < 0.0001). No significant change in blood pressure (p > 0.05). 123 patients (29%) responded to the satisfaction survey.  88% were very satisfied, and 9% quite satisfied as the DSN addressed their concerns.97% felt involved in treatment decisions.80% of respondents rated their post-intervention diabetes self-management as 8–10, with a mean score of 8.34, representing a 3-point increase from baseline.  78% reported making changes to diabetes self-care following the intervention." |
| **Ibrahim et al. 2019** | Impact of nurse-led clinic follow-up for post-PCI patients on 30-day mortality rate, readmission, and patient satisfaction. | 30 days re admission 30-day mortality, and patient satisfaction |  | **Satisfaction with the nurse-led clinic, Clinical Outcomes** | The study reported a 30-day mortality rate of 0.4% and a 30-day readmission rate of 10%, with only 1.8% due to cardiac reasons. Patient satisfaction was exceptionally high, with 98% of patients rating their experience as 10 out of 10 |
| **Kor et al.2022** | Explore clients' adherence and predictors to nursing recommendations and evaluate the clients' level of satisfaction with the nurse-led clinic. | Predictors of clients' adherence to nursing recommendations. | Level of satisfaction with the service | **Satisfaction with the nurse-led clinic, Knowledge about the disease** | Satisfaction rating – Very satisfied 78%, Satisfied- 17.2%. Lower level of education was a predictor of client nonadherence. |
| **Momoh et al.2024** | Exploring patient experiences and satisfaction with the new nurse-led diabetes clinic at BGH | Rating scores for various patient satisfaction parameters, including satisfaction with quality of care received, cleanliness/comfort, friendliness/compassion, time spent with clinical staff, diagnoses, treatment plans, usefulness of the advice received and confidence in following advice received. |  | **Satisfaction with the nurse-led clinic,** | Ratings: 100% for cleanliness and comfort. 94% for reasonable appointment wait time. 97% for convenience of appointment scheduling. 100% for recommending clinic to family or friends. 81% for willingness to join a diabetes support group. 98% for good quality of care. 99% for friendliness and compassion. 100% reported being satisfied with the time they spent with the clinic staff. 97% felt confident of following the agreed treatment plan with 3% not being confident of following the agreed plans. |
| **Nguyen et al. 2022** | To assess efficiency, time intervals. | Report impact of the RACPC on key timelines and patient satisfaction comparing a 6-month period prior to RACPC. | Patient satisfaction | **Timeliness of care** | Shorter time intervals from referral to clinic, 11 vs 83 days. Clinic to testing 18 vs 107 days. Satisfaction for those surveyed 98% rating overall service. |
| **Williams et al. 2012** | To assess patient satisfaction with the clinic’s ability to manage their postoperative needs. | The study aimed to assess patient satisfaction with the clinic’s ability to manage their postoperative needs | Assessing patient satisfaction and belief if their care would have benefited from seeing a doctor | **Acceptability** | In total, 73 (88%) patients believed their needs were met in the nurse-led clinic and regarding their overall satisfaction with the care and management received in the clinic, 82 (99%) patients described it as either good, very good or excellent.  In total, 73 (88%) patients believed their needs were met in the nurse-led clinic and regarding their overall satisfaction with the care and management received in the clinic, and 82 (99%) patients described it as either good, very good or excellent |
| **Winter et al.2012** | Assess patient satisfaction | Evaluate confidence in the advanced nurse practitioner, information provided, environment, running of the clinic |  | **Acceptability and Satisfaction with the nurse-led clinic** | 83% responded. 92% confidence in being managed by a nurse, 11% prefer a doctor. 95% satisfied with the information provided. 100% anxious about consultation. |

**Mixed Methods and Qualitative**

| **Author** | **Focus of study** | **Main outcome** | **Secondary outcome/s** | **Themes** | **Key Findings** |
| --- | --- | --- | --- | --- | --- |
| **Bala et al.202** | To describe how people with rheumatoid arthritis (RA) experience the care provided by Swedish nurse  Led rheumatology outpatient clinics. | Participants' experiences of care at a nurse-led clinic**.** |  | **Care & support** | The study identified three main categories describing  how the care offered by the nurse-led rheumatology  outpatient clinics were experienced: social environment,  professional approach, and value-adding care which provided a sense of security. Participants described the care received as professional and person-centred, with empathy, knowledge, and skill, which contributed to a positive experience. Also, nurses' specific knowledge and practical and teaching skills were instrumental for a positive experience. Also valued the continuity of care at the nurse led clinic |
| **Bennet-Daly et al.2021** | This study examines barriers to healthcare access amongst individuals who experience homelessness, | Examine barriers individuals who experience homelessness | client and staff perceptions of the MHNC services and explores opportunities for service expansion. | **Care & support** | Clients valued rapport, continuity of care, drop-in access, and fee-free services. The nurse-led model was highly appreciated, improved access, and reduced reliance on emergency departments. Barriers included gaps in services, stigma, and financial constraints**.** |
| **Gyldenvang et al.2022** | To explore patient satisfaction with nurse-led consultations but also to examine the experiences and perspectives of  clinical nurse specialists and physicians on the expanded scope of CNS’  nursing tasks and interdisciplinary teamwork | Patient satisfaction of the nurse led consultations was explored. Along with the experiences and perspectives of professionals on the expanded scope of CNS’ nursing tasks and  interdisciplinary teamwork |  | **Knowledge about the disease, Care & support,**  **Acceptability** | The patients agreed that they had received sufficient and appropriate support and knowledge to self-manage their side effects (89.8%) and were able to cope with their emotional reactions (68.8%) during and after chemotherapy. However, three patients (2.8%) said that getting a referral to speak with a physician upon request was limited.  Overall high levels of satisfaction with nurse-led consultations from the perspectives of patients with gynaecological- or breast cancer. Patients felt their needs were met and interactions were patient centred. |
| **Habibi et al.2023** | Experiences and feedback of adults with congenital heart disease who attended a nurse-led one-stop clinic. | The experiences and feedback from patients who attended the one-stop clinic to understand potential areas for service improvement. |  | **Knowledge about the disease, Acceptability** | The one-stop nurse-led clinic improved patient education, empowerment, and satisfaction by combining investigations and specialist consultations in a single visit |
| **Petrushnko et al.2024** | the purpose  of this study was to evaluate patient-reported satisfaction with  CCNC coordinated care, focusing on the early follow-up period. | Patient Satisfaction with the Colorectal cancer Nurse care |  | **Accessibility, Care & support,** | Over 90% of responders rated the colorectal cancer nurse's ability to coordinate care and provide information to patients and families as 'Very good' or 'excellent.' Additionally, 74% rated the nurse's professional conduct as 'excellent,' and 90% appreciated having the nurse as a single point of contact. Moreover, 86% felt they could easily contact the nurse when needed. Patients reported the care as excellent, highlighting attention to emotional needs, reduced stress, and increased confidence during treatment. |
| **Pun et al.2023** | This study explores the  experiences of patients with RA under nurse-led care, the roles that nurses play, and the outcomes achieved when applying the patient-centred care (PCC) approach. | Explore the experiences of Hong Kong RA patients and their perceptions of the nurse-led rheumatology clinic |  | **Accessibility, Acceptability Care & support** | Accessibility of RA nurses providing time to patients supported them emotionally: Seeing the same nurse provided continuity of care, nurses know more about  the characteristics of my disease symptoms". Increased medication compliance: "After learning more about the drugs from the nurse, I would  follow the instructions and take the correct amount of drugs. I  feel less pain in my joints now" |
| **Ramachandran et al.2021** | Experience of patients and medical staff of the nurse led clinic to identify the strengths and areas of improvement | Experiences of Patients in a Nurse-Led Cirrhosis Clinic | Views and experiences of hepatologists and SNS involved in the NLCCs | **Knowledge about the disease** | Personalised, non-judgmental care by the specialist nurse, longer and informative consultation sessions, accessible community setting, high satisfaction, confidence, and increased patient understanding |
| **Sjo and Bergsten 2018** | Evaluate patient's experiences with rheumatoid arthritis clinic | Describe the experience of patients with RA attending person‐centred, nurse‐led clinics over a 12‐month period. |  | **Acceptability, Knowledge about the disease** | Themes that emerged from this study included: encountering competence, Increased knowledge about the disease and treatment, Support from other healthcare professionals, experiencing participation, a sustainable relationship, an enhanced sense of security, easy access, making a personal journey, increased self-knowledge, and learning how to take care of myself. |
| **Stirling et al.2016** | To understand why people contact a NLMC and how their participation influences future planning can help us better target health care messages with the aim of improving health literacy | Client’s experiences of using the memory clinic, how participation affect client’s life, how did information provided impact on behaviour and understanding | . | **Acceptability** | NLMC was able to assess, diagnose, and make referrals for participants. Participants felt comfortable with the time available to discuss their concerns, valuing the opportunity to talk and be listened to. |
| **Taylor et al.2018** | To explore and describe lymphoma survivors’ thoughts and perceptions of the components of a nurse led  lymphoma survivorship clinic intervention. | Specific experience by the participants |  | **Care & support** | Participants described the reassurance they gained from having contact with a health professional post-treatment who individualised information and support. A survivorship care plan and treatment summary were developed for this study and was believed to be very patient-centred and helpful. This enabled participants to take back control of their health and well-being and to rebuild confidence. |
| **Vanalia et al.2023** | To assess patient  satisfaction with specialist nurse follow-up for patients with renal cancer  who have undergone surgery. | Patient satisfactions with consultations in primary care |  | **Care & support** | The audit revealed that in  terms of general satisfaction, 97.1% of patients felt totally satisfied with  their visit/consultation at the specialist nurse clinic. Furthermore, 94.2%  of patients felt that they had good continuity of care with specialist nurse  follow-up. |
