# Supplementary material for: Perceptions, perspectives and experiences of adult patients attending nurse-led clinics: a mixed-method systematic review
Source: BMC Nurs. 2026 May 20;25:620. doi: 10.1186/s12912-026-04740-7 (PMC13366822; doi:10.1186/s12912-026-04740-7)
Supplement: Supplementary file 5 — Supplementary material 5 [file 12912_2026_4740_MOESM5_ESM.docx]

**Appendix 5. GRADE-CERQual Findings**

| 1. *Bala, S. V., Samuelson, K., Hagell, P., Svensson, B., Fridlund, B., & Hesselgard, K. (2012). The Experience of Care at Nurse-Led Rheumatology Clinics. Musculoskeletal Care, 10(4), 202-211.* [*https://doi.org/10.1002/msc.1021*](https://doi.org/10.1002/msc.1021) | |
| --- | --- |
| *Findings 1.* | *Participants felt a sense of security and trust* ***(U)*** |
| *Illustration* | *I feel safe . . . I rely a lot on them! I think that what they say is good, and I try to take it in . . . they are very responsive and open to my illness*  *as well as to me as a person, and that is why I have so much confidence in them’ (p 208)* |
| *Illustration 2.* | *When I came here, I felt that here there was a very solid knowledge of rheumatology . . . the Knowledge was deep and meant that you got a*  *feeling of trust (p208)* |
| *Findings 2* | *Felt supported by the nurses, and is expressed as facilitating and simplifying their care and acted as a skilled advocate* ***(U)*** |
| *Illustration 1* | *You know that you can get help and that they always try to make it easier for you so that you get the contacts you are looking for’ (p208)* |
| 1. *Bennett-Daly, G., Unwin, M., Dinh, H., Dowlman, M., Harkness, L., Laidlaw, J., & Tori, K. (2021). Development and Initial Evaluation of a Nurse-Led Healthcare Clinic for Homeless and At-Risk Populations in Tasmania, Australia: A Collaborative Initiative. International Journal of Environmental Research and Public Health, 18(23), 12770.* [*https://doi.org/https://dx.doi.org/10.3390/ijerph182312770*](https://doi.org/https:/dx.doi.org/10.3390/ijerph182312770) | |
| *Findings* | *Participants felt trust, rapport, and continuity of care* ***(U)*** |
| *Illustration* | *They actually listen to me. They don’t judge you, like there’s all walks of life*  *that come here, but most of us are drug addicts, alcoholics or victims of one sort or the other”. (p8)* |
| *Illustration 2* | *Since I’ve been in Launceston for the last six months, I’ve been coming here to see the [nurses] every Thursday when I can”. (p9)* |
| 1. *Fishburn, A., & Fishburn, N. (2021). Establishing a nurse-led thyroid cancer Â follow-up clinic. British Journal of Nursing, 30(4), S28-S35.* [*https://doi.org/10.12968/bjon.2021.30.4.S28*](https://doi.org/10.12968/bjon.2021.30.4.S28) | |
| *Finding 1* | *Patients were extremely satisfied with the CNS-led clinic* ***(C)*** |
| *Illustration* | *I am very satisfied and pleased to be among the people who can say “thank you” for saving my life! I can’t imagine how you could improve on that! Grateful thanks’ (p33)* |
| 1. *Gyldenvang, H. H., Christiansen, M. G., Jarden, M., & Piil, K. (2022). Experiences and perspectives of patients and clinicians in nurse-led clinics in an oncological setting: A sequential multi-methods study. European Journal of Oncology Nursing, 61, 1-7.* [*https://doi.org/https://dx.doi.org/10.1016/j.ejon.2022.102203*](https://doi.org/https:/dx.doi.org/10.1016/j.ejon.2022.102203) | |
| *Findings 1* | *Participants were satisfied with skills of the CNS and the nurse led consultations* ***(U)*** |
| *Illustration 1* | *I experienced competent and forthcoming nurses who were prepared for each consultation and familiar with my treatment plan.” (p3)* |
| *Illustration 2* | *It was my first nurse-led consultation, and it was a very positive experience! The consultation was extremely well-prepared with relevant follow-up and questions. My medical record had been reviewed in advance and the most important aspects were clarified with a physician. A very positive experience!” (p3)* |
| 1. *Habibi, H., Poole, D., McDonnell, E., Lambell, M., Hipolito, A., Senko, B., Finlay, C., Haidu, L., Castro Meira, V., Doughty, V., De Sousa, S., Dimopoulos, K., & Gatzoulis, M. A. (2023). A nurse-led model: developing a one-stop clinic for patients with congenital heart disease. British Journal of Cardiac Nursing, 18(10), 1-11.* [*https://doi.org/10.12968/bjca.2023.0068*](https://doi.org/10.12968/bjca.2023.0068) | |
| *Findings* | *Patients felt CNCs gave clear explanations and showed a better understanding of their condition than previous providers, and accepted the nurse-led consultation* ***(U)*** |
| *Illustration 1* | *I felt that the [clinical nurse specialist] had a better day-to-day understanding of the condition and implications than I have had explained by a doctor****.’*** *(p6)* |
| *Illustration 2* | *All of the healthcare professionals who treated me were extremely friendly and warm and put me at ease. They did a great job of explaining the test to me.-- nurse appointment at the end was excellent and I have learned a lot.’ (p6)* |
| 1. *Pun, J., Tsang, C. M., Wong, J., & Kong, B. C. K. (2023). Experience of Patients With Rheumatoid Arthritis: A Qualitative Study of a Nurse-Led Clinic. Clinical Nursing Research, 32(4), 840-849.* [*https://doi.org/10.1177/10547738231164395*](https://doi.org/10.1177/10547738231164395) | |
| *Finding* | *Patient found nurse-led clinics are highly accessible with easy appointment scheduling* ***(C)*** |
| *Illustration* | *Sometimes [when I encounter things that are] unclear and when*  *I don’t know who to ask, I call the nurse; for example, to ask*  *about the drugs’ side effects. I don’t have to wait long. (p843)* |
| *Finding* | *Patients appreciated the psychological support provided by nurses, which facilitated their treatment compliance and improved their emotional well-being.* ***(U****)* |
| *Illustration 1* | *The services in the nurse-led clinic helped me a lot. They would*  *comfort me, which in turn helped with my medication use and*  *emotional stability. (p843)* |
| *Illustration 2* | *Nurses are able to explain our disease in detail, and they provide*  *us with emotional support in the nurse-led clinic, which makes*  *me happier. (p843)* |
| *Findings* | *Participants expressed confidence in the treatment they received from nursing professionals and considered nurses to be knowledgeable.* ***(U)*** |
| *Illustration1* | *Nurses not only help me and other patients, but also assist with*  *alleviating the doctors’ workload and stress. Nurse specialists*  *really are more than half a doctor (p844)* |
| *Illustration 2* | *My nurse is able to answer all my questions. For example, [my]*  *nurse could answer my questions about whether I will get sick*  *after taking the medication and my urinary tract infection*  *problem, and advised me on how to deal with and avoid these*  *situations. (p844)* |
| 1. *Ramachandran, J., Lawn, S., Tang, M. S. S., Pati, A., Wigg, L., Wundke, R., McCormick, R., Muller, K., Kaambwa, B., Woodman, R., & Wigg, A. (2022). Nurse Led Clinics; A Novel Model of Care for Compensated Liver Cirrhosis: A Qualitative Analysis. Gastroenterology Nursing, 45(1), 29-42.* [*https://doi.org/10.1097/SGA.0000000000000620*](https://doi.org/10.1097/SGA.0000000000000620) | |
| *Findings* | *Participants reported being happy with the way the information was provided in simple terms* ***(U)*** |
| *Illustration* | *I needed a lot of information and they explain it in layman’s terms.” (p35)* |
| 1. *Sjö, A. S., & Bergsten, U. (2018). Patients' experiences of frequent encounters with a rheumatology nurse—A tight control study including patients with rheumatoid arthritis. Musculoskeletal Care, 16(2), 305-312.* [*https://doi.org/10.1002/msc.1348*](https://doi.org/10.1002/msc.1348) | |
| *findings* | *Participants experienced nurses' competence gives them a sense of security.* ***(U)*** |
| *Illustration* | *…both the nurse and the doctor are highly specialized, so I never think that the nurse I'm meeting will be lacking in competence—that has never occurred to me… I know that if the nurse felt uncertain, she would check with a doctor, she wouldn't just chance it.(p308)* |
| *Findings* | *Having sufficient knowledge but not daring to ask questions led participants not to take medications as prescribed.* ***(U)*** |
| *Illustration* | *when I had had the opportunity to talk to her and pose all my questions… that is what has made me take the medications.(p308)* |
| 1. *Stirling, C., Campbell, B., Bentley, M., Bucher, H., & Morrissey, M. (2016). A qualitative study of patients' experiences of a nurse-led memory clinic. Dementia (London, England), 15(1), 22-33.* [*https://doi.org/https://dx.doi.org/10.1177/1471301213512841*](https://doi.org/https:/dx.doi.org/10.1177/1471301213512841) | |
| *Findings* | *Participants stated they would not have made an appointment with their GP to discuss their memory concerns, and they were pleased with the NP's assessment.****(C)*** |
| *Illustration* | *the doctor hasn’t got time . . . is a busy man (p26)* |
| 1. *Taylor, K., Monterosso, L., & Bulsara, C. (2018). Qualitative results from a phase II pilot randomised controlled trial of a lymphoma nurse-led model of survivorship care. European journal of oncology nursing: the official journal of European Oncology Nursing Society, 35, 9-14.* [*https://doi.org/https://dx.doi.org/10.1016/j.ejon.2018.01.011*](https://doi.org/https:/dx.doi.org/10.1016/j.ejon.2018.01.011) | |
| *Findings* | *Participants valued the one-on-one nurse-led intervention for providing reassurance, emotional support, and a safe space to discuss concerns beyond medical treatment.* ***(U)*** |
| *Illustration 1* | *“Just knowing that I was still going to get some support” (p11)* |
| *Illustration 2* | *They covered a multitude of different things like your emotional*  *well-being, mental well-being and physical well-being, all the things that*  *you know you can struggle with”(p11)* |
| *Illustration 3* | *Someone that you can speak to and address the problems that you don't*  *get the time with the doctors to talk about”(p11)* |
| *Illustration 4* | *And she did explain things so that I understood them more. She was*  *really good at making you feel relaxed” (p11)* |
| 1. *Winter, H., Lavenderb, V. T., & Blesingc, C. (2012). Assessing satisfaction with a nurse-led clinical trials clinic. Cancer Nursing Practice, 11(9), 23-27.* [*https://doi.org/10.7748/cnp2012.11.11.9.23.c9408*](https://doi.org/10.7748/cnp2012.11.11.9.23.c9408) | |
| *Findings* | *Satisfaction with the nurse-led clinic and the acceptability of the nurse led care* ***(C)*** |
| *Illustration 1* | *“The treatment and care I received was excellent”. “No criticism whatsoever from any aspect or person involved from beginning to end of my treatment”. (p11)* |
| *Illustration 2* | *“Throughout my treatment I was treated superbly with all the professional treatment anyone could ask for”. (p11)* |
